# The distribution of parent‐reported attention‐deficit/hyperactivity disorder and subclinical autistic traits in children with and without an ADHD diagnosis

**DOI:** 10.1002/jcv2.12223

**Published:** 2024-02-24

**Authors:** Tracey Chau, Jeggan Tiego, Louise E. Brown, Olivia J. Mellahn, Beth P. Johnson, Aurina Arnatkeviciute, Ben D. Fulcher, Natasha Matthews, Mark A. Bellgrove

**Affiliations:** ^1^ Turner Institute for Brain and Mental Health School of Psychological Sciences Monash University Clayton Victoria Australia; ^2^ School of Nursing, Midwifery & Paramedicine Curtin University Bentley Western Australia Australia; ^3^ School of Physics The University of Sydney Camperdown Western Australia Australia; ^4^ School of Psychology The University of Queensland Saint Lucia Queensland Australia

**Keywords:** attention‐deficit/hyperactivity disorder, factor mixture modelling, latent structure, subclinical autism

## Abstract

**Background:**

Autistic traits are often reported to be elevated in children diagnosed with attention‐deficit/hyperactivity disorder (ADHD). However, the distribution of subclinical autistic traits in children with ADHD has not yet been established; knowing this may have important implications for diagnostic and intervention processes. The present study proposes a preliminary model of the distribution of parent‐reported ADHD and subclinical autistic traits in two independent samples of Australian children with and without an ADHD diagnosis.

**Methods:**

Factor mixture modelling was applied to Autism Quotient and Conners' Parent Rating Scale – Revised responses from parents of Australian children aged 6–15 years who participated in one of two independent studies.

**Results:**

A 2‐factor, 2‐class factor mixture model with class varying factor variances and intercepts demonstrated the best fit to the data in both discovery and replication samples. The factors corresponded to the latent constructs of ‘autism’ and ‘ADHD’, respectively. Class 1 was characterised by low levels of both ADHD and autistic traits. Class 2 was characterised by high levels of ADHD traits and low‐to‐moderate levels of autistic traits. The classes were largely separated along diagnostic boundaries. The largest effect size for differences between classes on the Autism Quotient was on the Social Communication subscale.

**Conclusions:**

Our findings support the conceptualisation of ADHD as a continuum, whilst confirming the utility of current categorical diagnostic criteria. Results suggest that subclinical autistic traits, particularly in the social communication domain, are unevenly distributed across children with clinically significant levels of ADHD traits. These traits might be profitably screened for in assessments of children with high ADHD symptoms and may also represent useful targets for intervention.

**Key points**

**What's known?**

- Children with ADHD often also display subclinical autistic traits, but a baseline distribution of these traits in this population has not yet been established.

**What's new?**

- Using factor mixture modelling, two classes of participants largely separated along ADHD‐diagnostic boundaries were identified.
- This model also supports the conceptualisation of ADHD as a continuously distributed phenotype.
- Most children exhibiting clinically elevated levels of ADHD traits also displayed significantly higher levels of subclinical autistic traits. These traits were not uniformly elevated; instead, they were unevenly distributed in both samples. A large difference on the Autism Quotient's Communication scale was replicated across both samples.

**What's relevant?**

- There may be clinical utility in screening for subclinical autistic traits in children with ADHD, particularly if social communication difficulties are present.

## INTRODUCTION

Research supports the conceptualisation of attention‐deficit/hyperactivity disorder (ADHD) as the extreme end of a continuum of inattentive and hyperactive/impulsive traits (Posner et al., 2020; Sonuga‐Barke et al., 2023). Receiving co‐occurring psychiatric and/or neurodevelopmental diagnoses alongside an ADHD diagnosis is common and can substantially change the intervention pathway (Katzman et al., 2017). Indeed, when autism spectrum disorder (henceforth ‘autism’) traits co‐occur with ADHD traits, there are added complexities for assessment, intervention, service use, and outcomes (Antshel et al., 2016; Mellahn et al., 2022; Scandurra et al., 2019; Zablotsky et al., 2017).

Effects of co‐occurring autistic traits on cognitive and clinical symptomatology in children with ADHD have been found even at the subclinical level (e.g., Cooper et al., 2014; Grzadzinski et al., 2011), that is, where the traits are present and detectable but fall short of meeting diagnostic thresholds. These studies suggest that the presence of subclinical autistic traits can impact cognitive, social, and psychological wellbeing over and above any effects of existing ADHD symptoms (Cooper et al., 2014; Grzadzinski et al., 2011). As such, the identification of subclinical autistic traits may convey clinically meaningful information about how a child with ADHD interacts with the world around them. Given that autistic traits are also understood to exist on a continuum in the general population (Constantino & Todd, 2003), establishing a baseline distribution of subclinical autistic traits in individuals with ADHD may help to refine and optimise any recommendations for clinical decision‐making. Knowing this distribution could also have important implications for the predictive and discriminant validity of diagnostic tools (Antshel & Russo, 2019). Thus, expanding our understanding of the subclinical autistic trait phenotype in children with ADHD is an important step towards more tailored and accessible supports for neurodivergent children.

Studies attempting to model the relationship between ADHD and autism have employed a variety of methods, including latent class analysis and factor analysis (Farhat et al., 2022; Krakowski et al., 2021). Methodological differences in recruitment, measurement, and analysis mean that many results are only comparable to findings derived from analytical techniques of the same class. For example, Krakowski et al. (2021) highlighted in their scoping review that the latent class studies often identified more phenotypic overlap between autism and ADHD, whereas factor analytic studies tended to support distinct ‘autism’ and ‘ADHD’ factors. A key recommendation from their review was for future studies to employ statistical methods that combine multiple techniques together, such as factor mixture modelling (FMM). FMM allows for the detection of one or more classes of participants that differ along categorical and/or continuous latent variables. FMM is suitable for dimensional characterisations of data because it captures the variability between and within classes, thus facilitating the identification of potential subgroups that would not have otherwise been detected through separate latent class and factor analytic analyses (Clark et al., 2013).

Despite strong agreement that autistic traits are more prevalent in children with ADHD than in their neurotypical peers (e.g., Cooper et al., 2014; Green et al., 2015; Grzadzinski et al., 2011; Kochhar et al., 2011; Kotte et al., 2013; Okyar & Görker, 2020; Reiersen & Todd, 2008), only one study has investigated the latent structure of autistic traits in children with ADHD (Martin et al., 2014). Using exploratory factor analysis, Martin et al. (2014) found that the social and non‐social items of the Social Communication Questionnaire (SCQ) loaded onto distinct factors, consistent with findings in community samples. Secondary analyses of SCQ responses and 18 separate ADHD items revealed distinct social and inattentiveness factors, as well as a joint restricted/repetitive behaviour and hyperactivity factor. However, only one ADHD‐hyperactivity item loaded strongly onto this factor (*λ* = 0.50; Costello & Osborne, 2005), so it is possible that this result was an artefact of exploratory factor analysis (Krakowski et al., 2021). There is an opportunity to further explore autistic traits in children with and without ADHD using FMM. Such an approach could potentially identify one or more dimensions and latent (not directly observed) classes of autistic and ADHD trait presentations in children with and without ADHD.

Here we aim to establish a preliminary model of parent‐reported ADHD and subclinical autistic traits in children with and without formal ADHD diagnoses using FMM. To strengthen the validity of our findings (Bauer & Curran, 2004), analyses were undertaken in two independent datasets. First, we hypothesised that using factor analysis, caregiver‐reported subscales measuring ADHD and autistic traits would coalesce around two distinct factors: (1) ADHD and (2) Autism. Second, consistent with previous findings, we hypothesised that caregiver‐reported ADHD traits would form a continually distributed phenotype across those children with and without an ADHD diagnosis (Coghill & Sonuga‐Barke, 2012). Third, we predicted that FMM would recover two distinct classes embedded within distributed autistic and ADHD traits: (1) a class including predominantly neurotypical children and low caregiver‐reported ADHD traits; and (2) a class incorporating predominantly children diagnosed with ADHD with moderate‐to‐high caregiver‐reported ADHD traits. Based on previous latent class analyses of autistic and ADHD traits (e.g., van der Meer et al., 2012), we expected that autistic traits would be low in the first class and elevated in the second class, but their precise distribution in each class represented an exploratory part of our study.

## METHOD

### Research team

The authorship team consisted of both allistic and neurodivergent researchers with experience in neurodevelopment (T.C., J.T., L.B., O.M., B.P.J., N.M., M.A.B.), cross‐cultural psychology (T.C.), and computational psychiatry (A.A., B.F.). Our study adopted a participatory action approach (Bergold & Thomas, 2012); neurodivergent researchers provided the neurotypical lead and second authors with guidance regarding the presentation and interpretation of results and their implications, and the language used throughout the manuscript. Their feedback and perspectives significantly contributed to the understanding of the results within a real‐life context.

### Participants

#### Recruitment

Participants from the discovery sample were recruited through the Monash Autism‐ADHD Genetics and Neurodevelopment (MAGNET) project. Recruitment and test administration processes are described elsewhere (Knott et al., 2021).

Participants from the replication sample were recruited as part of a study that sought to better understand the biomarkers of ADHD with recruitment sites in Victoria and Queensland, Australia (see Matthews et al., 2012 for further information). While Matthews et al. (2012) employed a cut‐off score of 65 on the Conners' Parent Rating Scale‐Revised: Long Version for the ADHD group in their study, this study did not employ any cut‐off scores in order to capture a broader dimensional conceptualisation of observed and latent ADHD traits.

#### Inclusion and exclusion criteria

All participants underwent a case review either by a multidisciplinary team (discovery sample) or psychiatrist (replication sample). This involved a review of each study's relevant clinical measures to confirm existing diagnoses or the possible presence of new diagnoses (refer to Knott et al., 2021; Matthews et al., 2012 for a more detailed description of these procedures). Only children who were enrolled in either the (a) neurotypical, or (b) ADHD without co‐occurring autism groups, and whose diagnostic statuses were confirmed through these processes, were included as part of this study.

Children were excluded if they had received prior diagnoses of, or, through the case review process, were identified as meeting diagnostic criteria for a specific learning disorder, intellectual developmental disability, major depressive disorder, a psychotic disorder, specific language impairment, or another co‐occurring neurodevelopmental condition including autism.

### Ethical considerations

Written consent to participate was obtained from parents, while verbal assent was obtained from children. The MAGNET project was approved by Monash University Human Research Ethics Committee (CF16/1537–2016000806), Department of Education and Training Victoria Human Research Ethics Committee (2017_003570), and Monash Health Human Research Ethics Committee (RES‐19‐0000‐372A). The Biomarkers study was approved by the Royal Children's Hospital and The University of Queensland Human Research Ethics Committees.

### Materials

#### Demographic variables

Participating children's age, sex (male or female), and diagnostic status (ADHD or neurotypical) were collected during study enrolment.

#### Wechsler scales

Full‐scale intelligence (FSIQ) was estimated based on children's performances on either the Wechsler Preschool & Primary Scale of Intelligence – Fourth Edition (WPPSI‐IV; Wechsler, 2012), Weschler Intelligence Scale for Children – Fourth or Fifth Edition (WISC‐IV or WISC‐V; Wechsler, 2014), or Weschler Abbreviated Scale of Intelligence – Second Edition (WASI‐II; Wechsler, 2011) depending on their age.

#### Conners' Parent Rating Scale‐Revised: Long version (CPRS)

The CPRS (Conners et al., 1998) is a standardised, parent‐report screening questionnaire for ADHD in children and adolescents aged 3–17 years. Responses to the CPRS are summarised as *T*‐scores on 14 subscales that correspond to different features of ADHD and the DSM‐IV‐TR diagnostic criteria for the core ADHD traits (i.e., inattentiveness, hyperactivity, impulsiveness). Only the 5 summary scales (i.e., ADHD Index, CPRS Global Index, DSM‐IV Inattention, DSM‐IV Hyperactivity/Impulsivity, and DSM‐IV Total Score) were included in our analyses. The CPRS Global Index was chosen as it captures elements of psychopathology that are closely linked to the core symptom clusters of hyperactivity/impulsivity and inattentiveness, therefore enriching our understanding of the ADHD phenotype across the broader ADHD‐trait continuum (Conners, 1997). The ADHD Index also contains items that best distinguish between children with and without ADHD (Conners, 1997), which could be additionally helpful in differentiating between neurotypical and neurodivergent participants.

#### Autism spectrum quotient

Observable autistic traits were rated using either the AQ Child Version (AQ‐C; Auyeung et al., 2008) or the Adolescent AQ (AQ‐Adol; Baron‐Cohen et al., 2006) depending on the child's age. While there are some minor wording differences between the items that comprise each version of the AQ, the subscales are the same: they are the sum of items encompassing social skills, attention switching, attention to detail, communication, and imagination. As such, only the subscales were included in our analyses. As most participating parents completed the AQ‐C, both administered versions of the AQ were scored according to the AQ‐C scoring method (i.e., responses scored as either 0, 1, 2, or 3; Auyeung et al., 2008) to allow for direct comparison between age groups.

### Procedure

#### Test administration

The AQ, CPRS, and Wechsler scales were administered as part of the routine clinical and cognitive assessment test battery for all participating families. Parents typically completed the questionnaires while the children took part in other components of the studies. For families where children had been prescribed ADHD‐related medication (e.g., methylphenidate), parents were instructed to rate their child's behaviour while they were off medication. It was not expected that the presence or absence of ADHD‐specific medications would impact on the presentation of core autism characteristics (Sturman et al., 2017). Children were not required to withdraw from these medications while completing their cognitive assessment.

### Statistical analyses

Data were analysed using Mplus (Version 8.7) and IBM's SPSS Statistics (Version 27) and were screened and analysed for missingness. Multiple imputation was undertaken (Enders, 2010) using the fully conditional specification method (Van Buuren, 2007) only if data did not meet the assumptions for classification as missing at random or missing completely at random (MCAR). Otherwise, the missing data were considered to be ignorable (Little, 1988) and were not imputed.

As the MAGNET data were clustered by family, we used type ‘complex’ in Mplus to estimate all models based on our original sample as this accounts for the non‐independence of observations through the use of a Sandwich estimator for standard errors (Muthén & Muthén, 2017). Our subsequent analyses followed FMM guidelines outlined by Clark and colleagues (Clark et al., 2013) to determine the best fitting model for our data.

A one‐factor common factor model, confirmatory factor analysis (CFA), and latent profile analysis (LPA) were run to (a) establish comparison models and (b) to develop a priori upper bounds for the number of factors and classes estimated by FMM techniques respectively. CFA models based on the AQ and CPRS were derived based on existing support for the ability of the questionnaires to validly and reliably measure the latent constructs of autism and ADHD respectively (Auyeung et al., 2008; Conners et al., 1998). Discriminant validity was also analysed (see Appendix S1 in the Supporting Information for more details). Model fit for all CFA and LPA models were evaluated using conventional fit statistics with reference to suggested interpretations where available (Bagozzi & Yi, 2012). Post‐hoc modification indices were included in a second CFA model to improve model fit. Residual correlations were freely estimated as indicated by their (a) modification indices, (b) consistency with theory, and (c) statistical significance when corrected for multiple comparisons using the Benjamini‐Hochberg procedure (Benjamini & Hochberg, 1995).

FMM analyses commenced with the one‐factor, one‐class models followed by the one‐factor, two class models. The number of classes and factors were increased up to their respective upper bounds as specified by previous analyses. All 4 main FMM variations were derived where possible (denoted FMM‐1 through FMM‐4; see Clark et al., 2013 for a more detailed explanation). We began with the most restrictive model (i.e., FMM‐1) where all parameters were held invariant across classes except for the factor means, and progressed sequentially through each model variation where equality constraints were relaxed on the factor variance‐covariance matrix (FMM‐2), intercepts (FMM‐3), and lastly the factor loadings (FMM‐4). Recommendations by Nylund, Asparouhov and Muthén (2007), endorsed by Clark et al., 2013, were followed to determine the model with the optimal number of factors and classes (refer to Appendix S2 for more information). The chosen model was then re‐run with age and FSIQ included as auxiliary variables using the BCH procedure (Asparouhov & Muthén, 2020) to investigate whether there were any differences between classes on those variables.

Each participant's probabilistic class assignment (i.e., the class that they were most likely to be allocated to based on their AQ and CPRS ratings) was also saved. Where means of FMM‐derived latent variables could not be compared (i.e., FMM‐3 or FMM‐4), a multivariate analysis of variance (MANOVA) was run to investigate whether there were any class‐based differences in AQ and CPRS subscale ratings. This classify‐analyse approach can be implemented when class separation is high (Entropy ≥0.80), such that class membership can be used as a discrete categorical variable (Clark & Muthén, 2009). Where homogeneity of error variances (i.e., Levene's Test) could not be assumed, Brown‐Forsythe tests (Brown & Forsythe, 1974) were run and the *p*‐values were evaluated against Benjamini–Hochberg corrected alpha levels for a false discovery rate of 0.001 (Benjamini & Hochberg, 1995).

## RESULTS

### Discovery sample – MAGNET data

A total of *N* = 208 (*n*_Neurotypical_ = 169, *n*_ADHD_ = 39) children enrolled in the MAGNET project met the inclusion criteria for this study. Participants were aged between 6 and 15 years (*M* = 8.21 years, *SD* = 2.77 years) and groups were equivalent in age (*t*(73.055) = −1.686, *p* = 0.096). There were approximately equal numbers of males and females in the sample (52.9% female, *n* = 110). Females were more likely to be in the neurotypical group (*χ* ^2^(1, *N* = 208) = 4.007, *p* = 0.045), but with a weak effect size (Cramer's *V* = 0.139; adapted from Cohen, 1988). Proportion of missing data was estimated at 9.28%. Little's MCAR Test was not significant (*χ* ^2^(46, *N* = 208) = 55.513, *p* = 0.159), indicating that data missingness was ignorable (Little, 1988). All scores for the continuous variables were within the expected upper and lower bounds of each measure (see Table S1 for descriptive statistics). Most of the continuous variables deviated significantly from normality (see Table S2). Given the non‐normality and missingness of the data, maximum likelihood with robust standard errors (MLR) estimation was used for subsequent analyses (Muthén & Muthén, 2017).

Modelling results

Here we summarise the model fit statistics from the one‐factor common factor model, CFA, LPA, and FMM analyses in our discovery sample (Table 1). BLRT*p* values were not available for this sample due to the clustered nature of the data.

**TABLE 1:** Discovery sample: Factor analysis, latent profile analysis, and factor mixture modelling results.

Model	Log likelihood	Entropy	AIC	BIC	VLMR*p*
Common factor Model					
1 factor	−5645.404	‐	11868.053	11957.643	‐
Confirmatory factor analysis					
2 factor	−5645.404	‐	11510.280	11424.726	‐
2 factor (modified)	−5645.404	‐	11603.187	11524.269	‐
Latent profile analysis					
Equal variances across classes					
1 class	−8008.480	‐	16056.961	16123.711	‐
2 classes	−6138.171	0.966	12332.342	12425.250	0.000
3 classes	−5979.992	0.944	12035.983	12162.072	0.005
4 classes	−5910.513	0.930	11917.026	12076.296	0.360
5 classes	−5848.692	0.933	11813.384	12005.835	0.096
6 classes	−5801.722	0.927	11739.443	11965.076	0.765
Freely estimated variances across classes					
1 class	−8008.480	‐	16056.961	16123.711	‐
2 classes	−6063.392	0.966	12200.783	12323.554	0.033
3 classes	−5880.020	0.944	11872.040	12057.855	0.122
4 classes	−5781.202	0.946	11712.403	11961.262	0.377
5 classes	−5713.010	0.937	11614.020	11925.923	0.260
6 classes	−5644.289	0.945	11514.579	11889.526	0.241
Factor mixture modelling					
1 factor, 2 classes					
FMM‐1	−6138.171	0.966	12332.342	12425.250	0.000
FMM‐2	−5891.134	0.746	11840.267	11936.493	0.007
FMM‐3	−5766.425	0.840	11606.849	11729.620	0.165
FMM‐4	−5757.401	0.882	11604.803	11754.118	0.001
1 factor, 3 classes					
FMM‐1	−5984.705	0.941	12029.410	12128.954	0.002
2 factors, 2 classes					
FMM‐1^a^					
FMM‐2	−5688.251	0.845	11444.503	11557.319	0.003
**FMM‐3**	**−5648.578**	**0.915**	**11379.157**	**11515.200**	**0.002**
FMM‐4^a^					
2 factors, 3 classes					
FMM‐1^a^					

*Note*: **Bolded** = best fitting model.

Abbreviations: AIC, Akaike Information Criterion; BIC, Bayesian Information Criterion; FMM‐1 model, freely estimates class factor means; FMM‐2 model, freely estimates class factor means and the factor covariance matrix; FMM‐3 model, freely estimates class factor means, the factor covariance matrix, and item thresholds; FMM‐4 model, freely estimates class factor means, the factor covariance matrix, item thresholds, and factor loadings; VLMR*p*, Vuong‐Lo‐Mendell‐Rubin *p*‐value.

^a^ Model misspecified.

#### CFA

Two CFA models were obtained: (1) an original model, and (2) a modified model with the inclusion of post‐hoc modifications (see Figures S1‐S2 for more details). As outlined in Table 1, Model 2 fit the data better than model 1 across all goodness of fit indices (Bagozzi & Yi, 2012). Although the chi‐square value was significant in both models, investigation of the residual correlation matrices of both models (see Tables S3‐S4) indicated that the bivariate relationships between variables were more accurately reproduced in Model 2 than Model 1. Furthermore, all correlation residuals in model 2 were ≤0.1, indicating that the model closely reproduced the bivariate correlations between the variables (acceptable local fit; Kline, 2015). Standardised and unstandardised factor loading coefficients for both models can be found in Table S5. The 95% confidence interval for the factor intercorrelation did not contain 1.00, and the squared correlation was 47.3% while the average variance extracted was 58.3% and 85.6% for the autistic and ADHD traits factors, respectively. These results indicate discriminant validity between our two factors (Fornell & Larcker, 1981; Hair, 2014).

#### LPA

Based on the BIC and VLMR*p* values displayed in Table 1, the ideal class upper bound was 3 for models with equal variance across classes (i.e., FMM‐1) and 2 for models with freely estimated variances across classes (i.e., FMM‐2, FMM‐3, FMM‐4).

#### FMM

Models with 1 and 2 factors and up to 3 classes were fit based on the CFA and LPA results. The 2‐factor, 2‐class FMM‐3 model was chosen as the best‐fitting model as it had the lowest BIC value and the VLMR*p*‐value was <0.05. The FMM model was strongly supported in favour of the modified CFA model based on the Bayes factor (BF = 93.18; Kass & Raftery, 1995).

As the model demonstrated acceptable class separation (entropy >0.80; Greenbaum et al., 2005), further analyses at the class‐level were conducted. Seventy‐nine percent of children in the ADHD‐only group (*n* = 30 of 38) were allocated to Class 2 while 74% of children in the neurotypical group were allocated to Class 1 (*n* = 123 of 166), indicating observable separation by diagnostic boundaries.

A summary of the auxiliary variable analyses can be found in Tables S6‐S7. Children in Class 1 were significantly older (*χ* ^2^(1, *N* = 208) = 4.23, *p* = 0.040), and had higher mean FSIQ (*χ* ^2^(1, *N* = 208) = 4.66, *p* = 0.031), than participants in Class 2. However, when considered against the Wechsler index score metrics (*M* = 100, *SD* = 15), both means fell comfortably around the overall mean and class means differed by less than one standard deviation. Additional chi‐square analyses indicated that females were significantly more likely to be allocated to Class 1 (*χ* ^2^(1, *N* = 208) = 9.055, *p* = 0.003) with a small‐to‐moderate effect size (Cramer's *V* = 0.211; adapted from Cohen, 1988).

Table 2 displays the mean AQ and CPRS subscale scores by class. Participants in Class 2 tended to be rated higher on both the AQ and CPRS than participants in Class 1. The mean CPRS scores for participants in Class 1 all fell within the ‘Average’ range (i.e., concerns are not clinically significant) while all scores for participants in Class 2 fell within the ‘Very Elevated’ range (i.e., concerns are clinically significant; Conners et al., 1998).

**TABLE 2:** Discovery sample: Summary of AQ and CPRS subscale scores by class assignment.

	Class 1	Class 2
	M (SD)	M (SD)
AQ subscales (standardised)		
Social skill	−0.247 (0.91)	0.440 (1.00)
Attention switching	−0.386 (0.89)	0.688 (0.80)
Attention to detail	−0.252 (0.88)	0.448 (1.05)
Communication	−0.401 (0.87)	0.714 (0.81)
Imagination	−0.256 (0.96)	0.456 (0.91)
CPRS scores		
ADHD index	53.89 (11.74)	73.63 (10.26)
Global index	52.11 (10.79)	76.49 (9.75)
DSM‐IV inattention	54.32 (12.53)	71.59 (11.61)
DSM‐IV hyperactivity/Impulsivity	51.34 (7.43)	79.04 (8.14)

*Note*: Standardised AQ scores are presented in this table.

MANOVA results comparing the mean subscale scores of participants between classes can be found in Table S8. These results are briefly described here. Box's *M* was non‐significant (*p* = 0.096), indicating that multivariate homogeneity of variance could be assumed. At the univariate level, results from Levene's test indicated the homogeneity of error variance assumption held for all subscales except for the AQ Attention to Detail subscale (*F*_mean_ = 5.773, *p* = 0.017). The Brown‐Forsythe test on this subscale showed a large significant effect of class assignment. Large and significant multivariate (Pillai's trace = 0.797, *F*(9, 193) = 84.437, *p* < 0.001, partial *η* ^2^ = 0.797) and medium‐to‐large significant univariate effects of class assignment were detected across all other subscales. The largest effects on the AQ subscales were detected for the Attention Switching (partial *η* ^2^ = 0.267) and Communication (partial *η* ^2^ = 0.288) subscales (refer to Table S8). Figures 1 and 2 display histograms of the distribution of AQ and CPRS subscale score frequencies by class assignment.

### Replication sample – Biomarkers of ADHD study

FIGURE 1Discovery sample: Histogram of AQ subscale score frequencies by factor mixture modelling class assignment.

**Figure:**
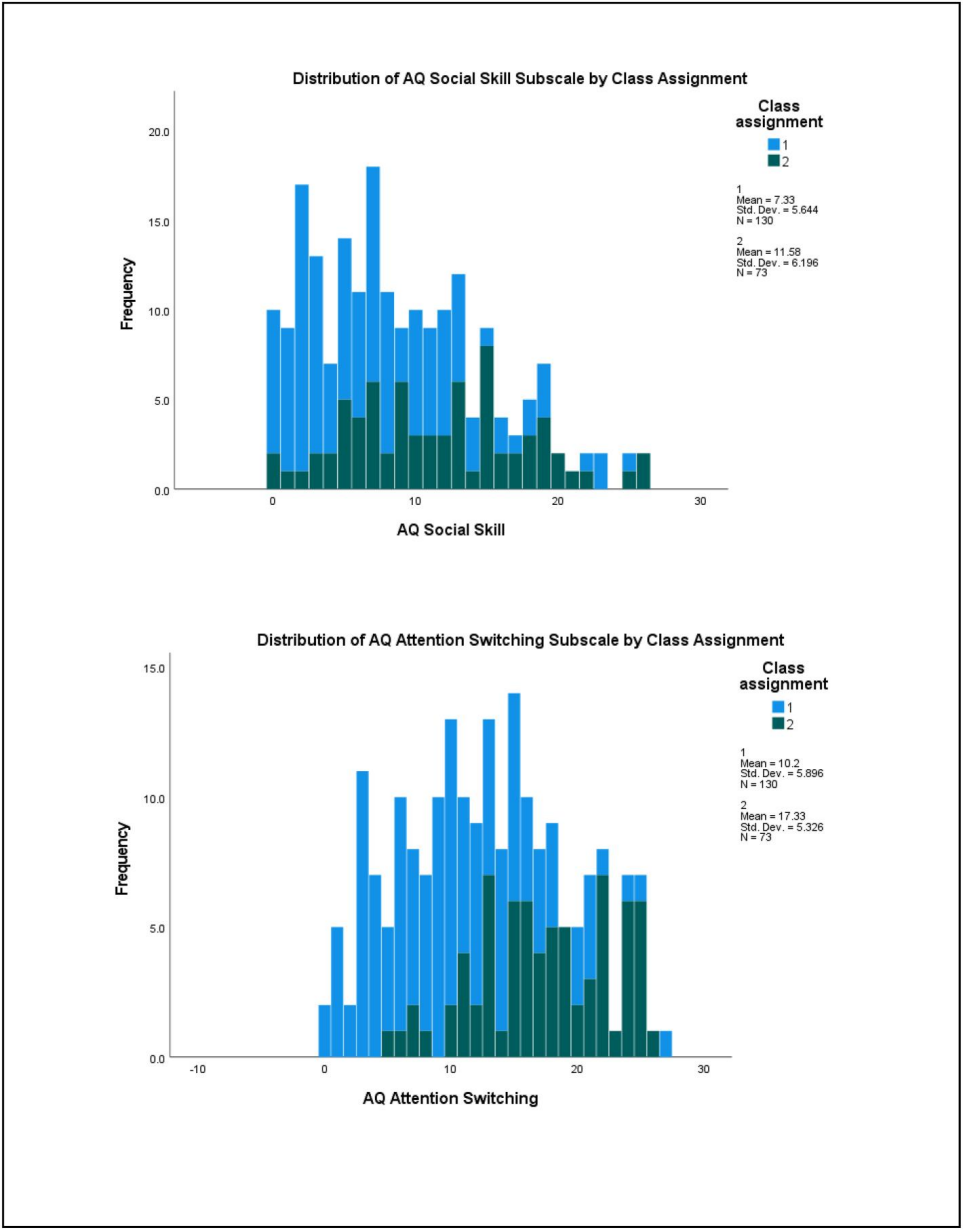

**Figure:**
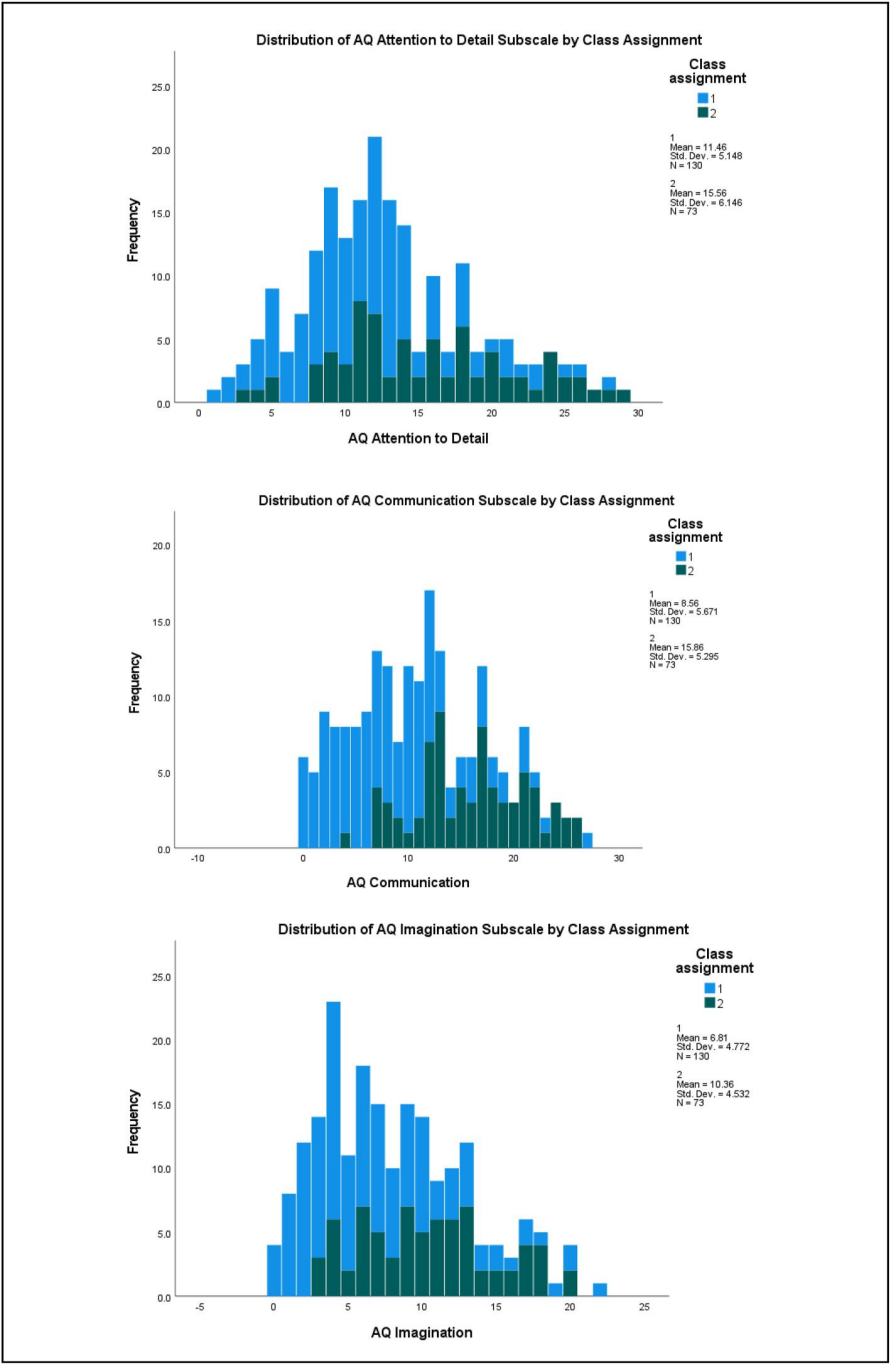

FIGURE 2Discovery sample: Histogram of CPRS subscale score frequencies by factor mixture modelling class assignment.

**Figure:**
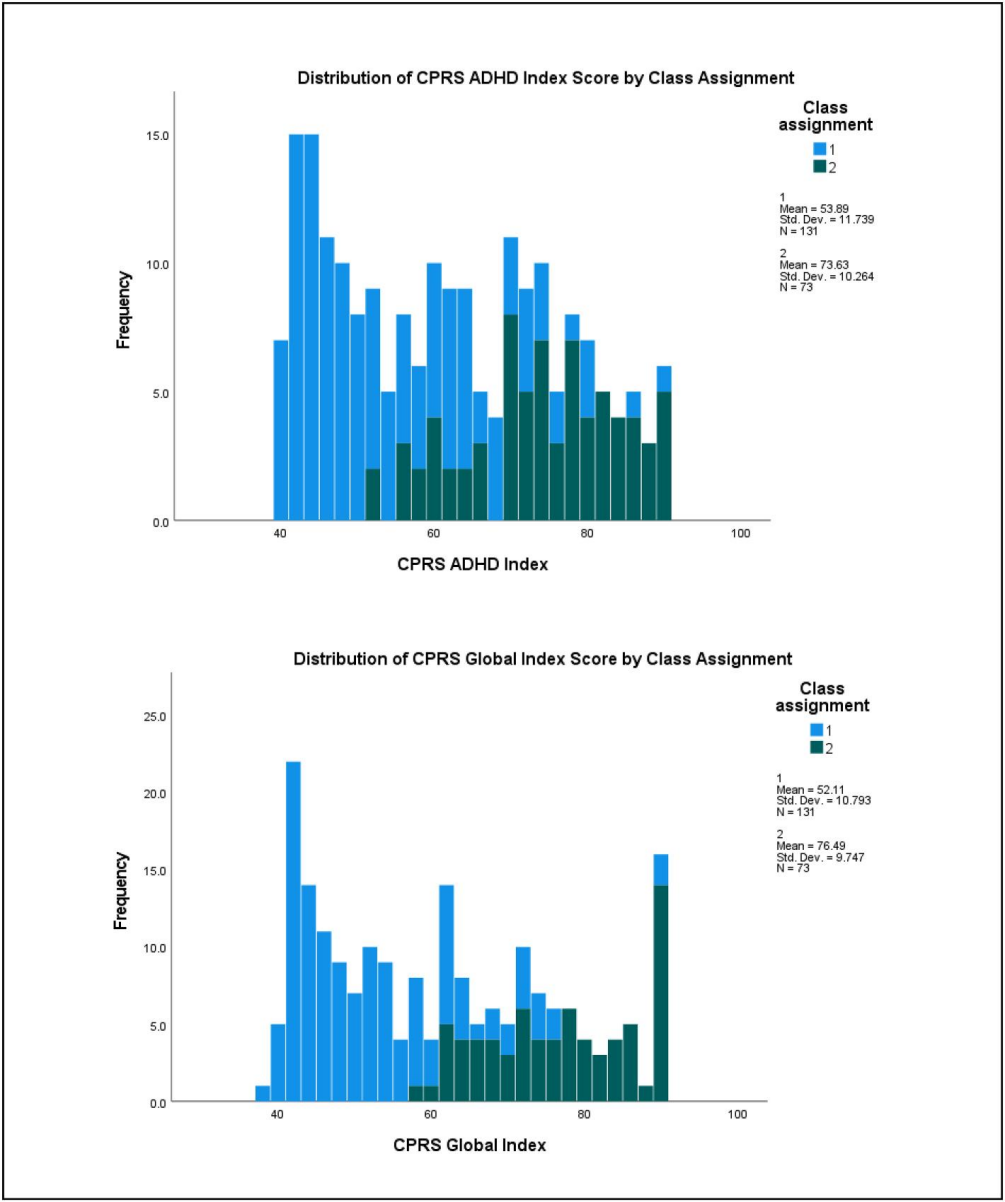

**Figure:**
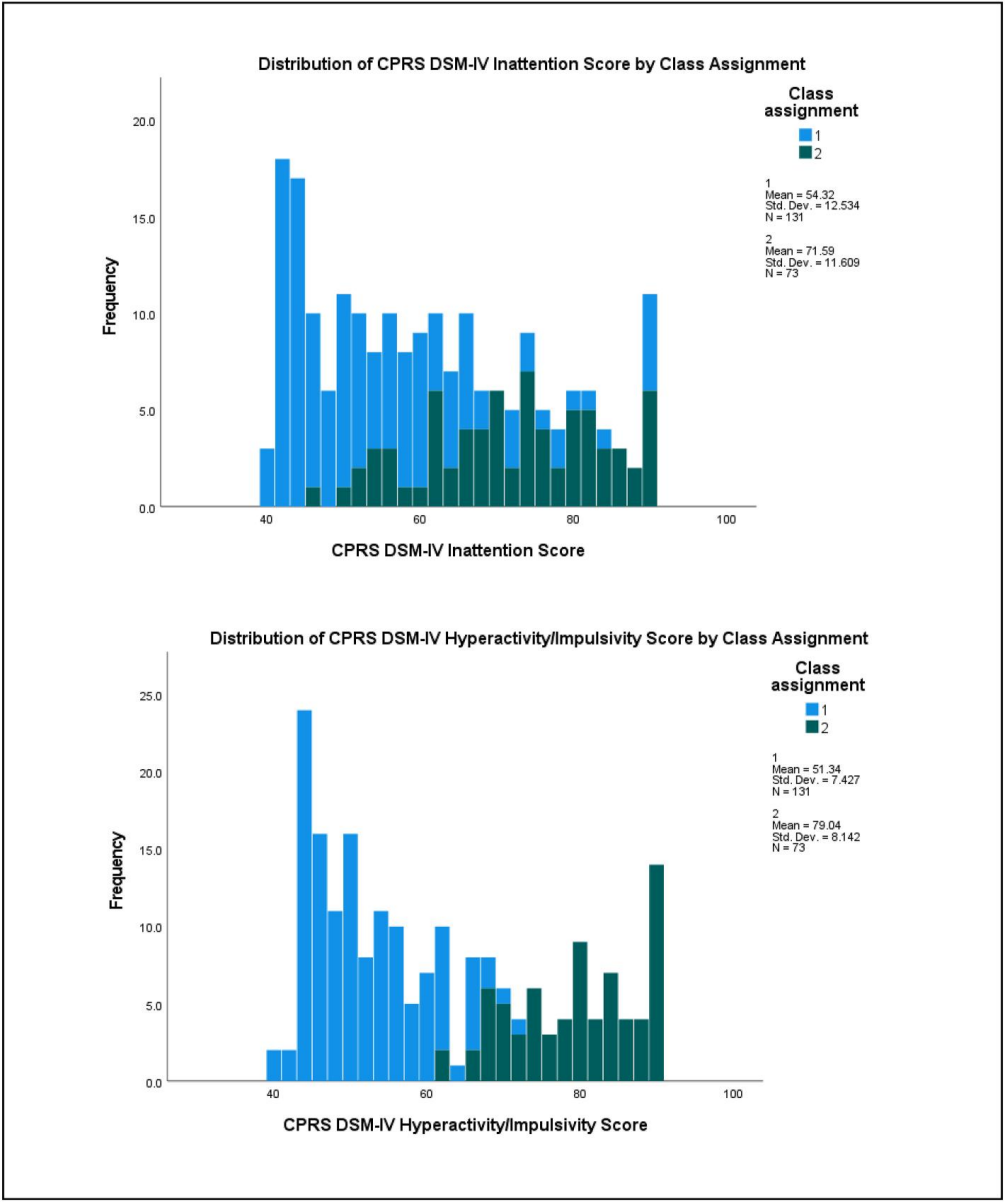

A total of *N* = 385 (*n*_Neurotypical_ = 280, *n*_ADHD_ = 105) children enrolled in the Biomarkers study met the inclusion criteria for this study. Three participants were excluded as they did not complete any component of the study visit (and thus, no data were available), leaving a final sample size of *N* = 382 (*n*_Neurotypical_ = 278, *n*_ADHD_ = 104). Participants were aged between 6 and 15 years (*M* = 10.93 years, *SD* = 2.15 years) and children in the ADHD group were significantly younger than neurotypical participants (*M*_Neurotypical_ = 11.19 years, *M*_ADHD_ = 10.23; *t*(380) = 3.982, *p* < 0.001). There were more males than females in the sample (26.8% female, *n* = 103). Males were significantly more likely to be in the ADHD group (χ^2^(1, *N* = 382) = 4.339, *p* = 0.037), though the effect size was small (Cramer's *V* = 0.107; adapted from Cohen, 1988).

Data could not be assumed to be missing completely at random (8.01% missing data; Little's Test: χ^2^(48, *N* = 406) = 84.758, *p* = 0.001; Little, 1988). As such, the data were imputed with multiple imputation, and the pooled dataset was used for subsequent analyses.

Descriptive statistics for the continuous variables after imputation are displayed in Table S9. Data remained non‐normal after imputation (see Table S10). All scores were within the specified bounds of each measure.

Modelling results

Here we summarise the model fit statistics from the one‐factor common factor model, CFA, LPA, and FMM analyses in our replication sample (Table 3).

**TABLE 3:** Replication sample: Factor analysis, latent profile analysis, and factor mixture modelling results.

Model	Log likelihood	Entropy	AIC	BIC	VLMR*p*	BLRT*p*
Common factor Model						
1 factor	−9924.343	‐	22062.351	22168.877	‐	
Confirmatory factor analysis						
2 factor	−9924.343	‐	20144.373	19992.358	‐	‐
2 factor (modified)	−9924.343	‐	20254.845	20130.447	‐	‐
Latent profile analysis						
Equal variances across classes						
1 class	−12350.210	‐	24106.420	24177.438	‐	‐
2 classes	−10988.997	0.988	22033.994	22144.466	0.000	0.000
3 classes	−10746.316	0.980	21568.633	21718.559	0.011	0.000
4 classes	−10506.950	0.936	21109.900	21299.280	0.424	0.000
5 classes	−10333.807	0.945	20783.614	21012.448	0.145	0.000
6 classes	−10246.790	0.950	20629.580	20897.868	0.046	0.048
Freely estimated variances across classes						
1 class	−12035.210	‐	24106.420	24177.438	‐	‐
2 classes	−10730.366	0.961	21534.731	21680.712	0.000	0.000
3 classes	−10336.959	0.948	20785.918	21006.862	0.023	0.000
4 classes	−10082.317	0.963	20314.634	20610.540	0.002	0.000
5 classes	−9908.115	0.963	20004.230	20375.099	0.138	0.000
6 classes	−9783.572	0.967	19793.145	20238.977	0.119	0.000
Factor mixture modelling						
1 factor, 2 classes						
FMM‐1	−10988.997	0.988	22033.994	22144.466	0.000	0.000
FMM‐2	−10536.568	0.932	21131.136	21245.553	0.000	0.000
FMM‐3	−10225.894	0.891	20525.788	20671.769	0.000	0.000
FMM‐4	−10195.500	0.888	20481.000	20658.544	0.000	0.000
1 factor, 3 classes						
FMM‐1	−10764.232	0.970	21588.464	21706.827	0.173	0.000
FMM‐2	−10504.961	0.927	21071.923	21194.231	0.240	1.000
FMM‐3	−10068.032	0.897	20230.064	20415.499	0.473	0.000
FMM‐4	−10024.885	0.890	20175.769	20424.331	0.108	0.000
1 factor, 4 classes						
FMM‐1	−10617.664	0.935	21299.329	21425.582	0.008	0.500
FMM‐2	−10499.306	0.891	21064.611	21194.810	0.413	1.000
FMM‐3	−9968.777	0.884	20051.554	20276.443	0.182	0.000
FMM‐4^b^	−9891.909	0.908	19945.818	20265.397	0.081	0.000
1 factor, 5 classes						
FMM‐1	−10547.633	0.934	21163.267	21297.411	0.397	0.500
FMM‐2	−10487.185	0.871	21044.370	21182.460	0.785	1.000
FMM‐3	−9893.407	0.935	19920.815	20185.158	0.629	0.000
FMM‐4^a^						
1 factor, 6 classes						
FMM‐1	−10521.616	0.936	21115.232	21257.267	0.393	1.000
FMM‐2	−10474.066	0.898	21022.132	21168.112	0.019	1.000
FMM‐3	−9827.893	0.929	19809.786	20113.583	0.218	0.000
FMM‐4^b^						
2 factors, 2 classes						
FMM‐1^b^						
FMM‐2	−9882.546	0.801	19833.092	19967.236	0.240	0.000
FMM‐3	**−9846.378**	**0.902**	**19774.755**	**19936.518**	**0.000**	**0.000**
FMM‐4^b^						
2 factors, 3 classes						
FMM‐1^b^						
FMM‐2	−9848.840	0.784	19777.680	19935.497	0.003	0.000
FMM‐3^b^						
FMM‐4^b^						
2 factors, 4 classes						
FMM‐1^b^						
FMM‐2^b^						
FMM‐3^b^						
FMM‐4^b^						
2 factors, 5 classes						
FMM‐1^b^						
FMM‐2^b^						
FMM‐3^b^						
FMM‐4^b^						
2 factors, 6 classes						
FMM‐1^b^						
FMM‐2^b^						
FMM‐3^a^						
FMM‐4^b^						

*Note*: **Bolded**, best fitting model.

Abbreviations: AIC, Akaike Information Criterion; BIC, Bayesian Information Criterion; BLRT*p*, Bootstrapped Likelihood Ratio Test *p*‐value; VLMR*p*, Vuong‐Lo‐Mendell‐Rubin *p*‐value.

^a^ Loglikehood was not replicated.

^b^ Model misspecified.

#### CFA

Once again, two CFA models were obtained: (1) the original model and (2) a modified model (see Figures S3‐S4). Investigation of model (2)'s residual correlation matrix (see Tables S11‐S12 for both models' matrices) indicated that all bivariate relationships were adequately reproduced (all values < |0.1|; Kline, 2015). On a balance of all other fit statistics in Table 3, adequate‐to‐good model fit remained supported for model (2) (Bagozzi & Yi, 2012). Coefficients for both models are summarised in Table S13. Once again, the 95% confidence interval for the factor intercorrelation did not contain 1.00, and squared correlation (33.5%) was lower than the average variance extracted for the autistic (65.3%) and ADHD (88.7%) trait factors. These results suggested discriminant validity between our two factors (Fornell & Larcker, 1981; Hair, 2014).

#### LPA

Based on the BIC and VLMR*p* values displayed in Table 4, the ideal upper bound for the number of classes was 6 for all FMM models.

**TABLE 4:** Replication sample: Summary of AQ and CPRS subscale scores by class assignment.

	Class 1	Class 2
	M (SD)	M (SD)
AQ Subscales (standardised)		
Social skill	−0.356 (0.697)	0.741 (1.12)
Attention switching	−0.271 (0.856)	0.563 (1.05)
Attention to detail	−0.068 (0.970)	0.142 (1.05)
Communication	−0.418 (0.658)	0.871 (1.03)
Imagination	−0.295 (0.791)	0.614 (1.11)
CPRS Scores		
ADHD index	45.51 (4.28)	71.06 (9.85)
Global index	45.98 (5.05)	70.11 (12.01)
DSM‐IV inattention	45.95 (5.09)	70.25 (10.45)
DSM‐IV hyperactivity/Impulsivity	47.43 (4.93)	73.26 (12.76)

*Note*: Standardised AQ scores are presented in this table.

#### FMM

Models with 1 and 2 factors and up to 6 classes were fit based on the CFA and LPA results. We began with the model that had the lowest BIC value: the 2‐factor, 3‐class FMM‐2 model. The VLMR*p*‐value for this model was <0.05, favouring this model over the *k*‐1 class models. However, there was only a one‐point difference between this model and the model with the next lowest BIC value (i.e., the 2‐factor, 2‐class FMM‐3). Bayes' factor comparison was very weak (BF = 1.66; Kass & Raftery, 1995), therefore favouring the more parsimonious 2‐factor, 2‐class FMM‐3 model. The competing model also demonstrated higher entropy, lower AIC, and a lower loglikelihood value. Thus, on a balance of all fit statistics, parsimony, and its substantive interpretability, the 2‐factor, 2‐class FMM‐3 model was chosen as the best fitting model. This FMM model was strongly favoured over the best‐fitting CFA and LPA models (BF > 100).

Once again, further class‐level analyses were conducted as class separation was good (entropy >0.80; Greenbaum et al., 2005). Ninety‐six percent of children in the ADHD‐only group (*n* = 100 of 104) were allocated to Class 2 while 91% of children in the neurotypical group were allocated to Class 1 (*n* = 254 of 278), indicating close separation along diagnostic boundaries.

A summary of the auxiliary variable analyses can be found in Tables S14‐S15. Children in Class 1 were significantly older (*χ* ^2^(1, *N* = 382) = 18.34, *p* < 0.001) and had higher mean FSIQ scores than children in Class 2 (*χ* ^2^(1, *N* = 382) = 60.87, *p* < 0.001). The difference in FSIQ remained below 1 standard deviation according to Wechsler index metrics (*M* = 100, *SD* = 15). Subsequent chi‐square analyses demonstrated no significant sex differences between classes (*χ* ^2^(1, *N* = 382) = 2.510, *p* = 0.113).

Table 4 displays the mean AQ and CPRS subscale scores by class for our replication sample. Consistent with results from our discovery sample, participants in Class 2 tended to be rated higher on both the AQ and CPRS than participants in Class 1. The mean CPRS scores for participants in Class 1 all fell within the ‘Average’ range (i.e., concerns are not clinically significant) while all scores for participants in Class 2 fell within the ‘Very Elevated’ range (i.e., concerns are clinically significant; Conners et al., 1998).

The MANOVA results comparing the mean subscale scores of participants between classes are summarised here (refer to Table S16 for more details). As Box's *M* was significant (*p* < 0.001), Pillai's Trace is reported as it is robust to violations of multivariate homogeneity of variance assumptions (Tabachnick & Fidell, 2013). Large and significant multivariate effects of class assignment were detected (Pillai's trace = 0.798, *F*(9, 372) = 163.533, *p* < 0.001, partial *η* ^2^ = 0.798). However, at the univariate level, results from Levene's test indicated that the homogeneity of error variance assumption did not hold for any subscales except for the AQ's Attention to Detail subscale (*F*_mean_ = 1.387, *p* = 0.240; *F*_median_ = 1.445, *p* = 0.230), although there were no significant differences between classes on this variable (*F*(1, 380) = 3.729, *p* = 0.054). As such, univariate Brown–Forsythe tests were undertaken with the remaining variables. There was a significant difference between groups on all variables at a false discovery rate of 0.001, with the largest effect on the AQ subscales found for the Communication (partial *η* ^2^ = 0.365) and Social Skill (partial *η* ^2^ = 0.265) subscales (see Table S16 for full results). Figures 3 and 4 display histograms of the distribution of AQ and CPRS subscale score frequencies by class assignment.

FIGURE 3Replication sample: Histogram of AQ subscale score frequencies by factor mixture modelling class assignment.

**Figure:**
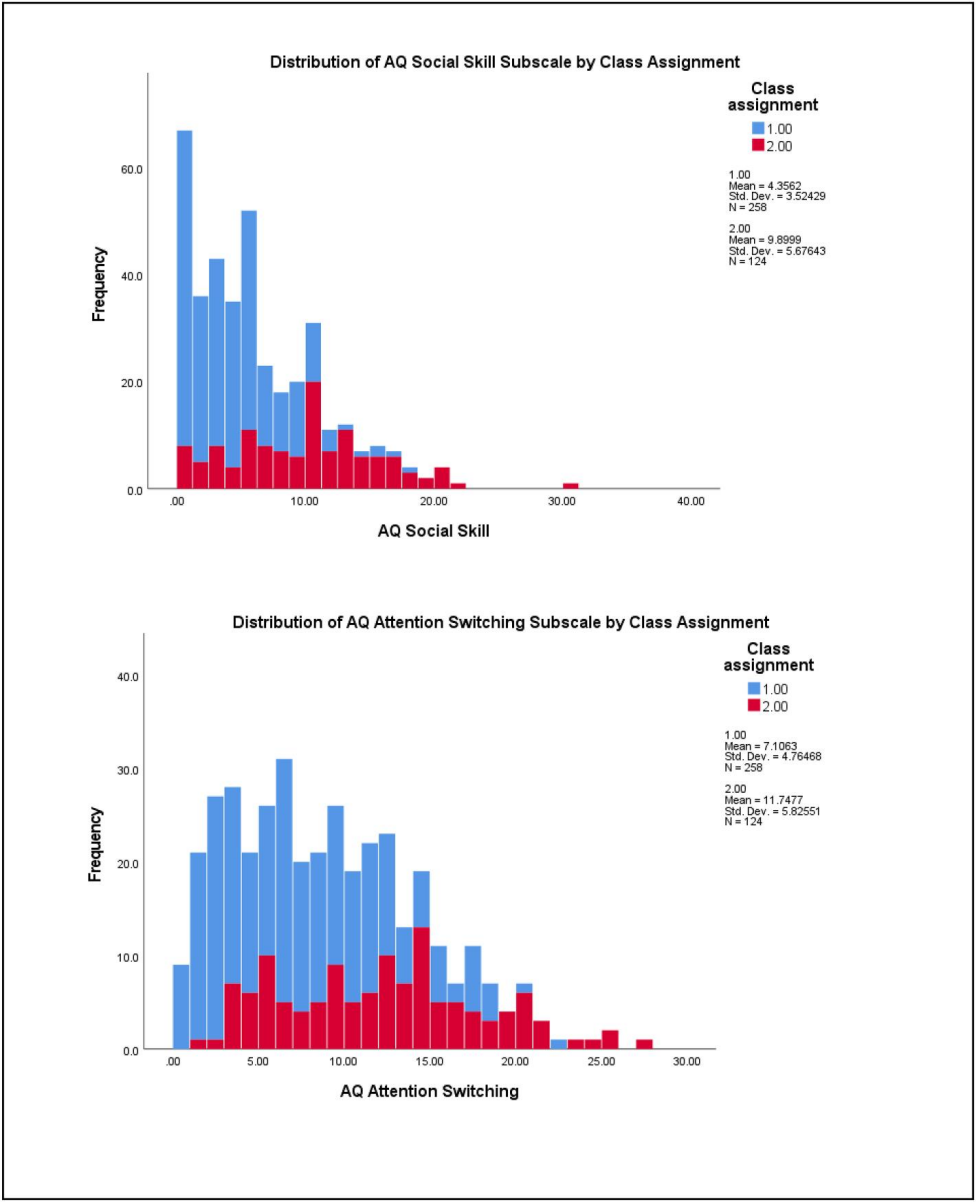

**Figure:**
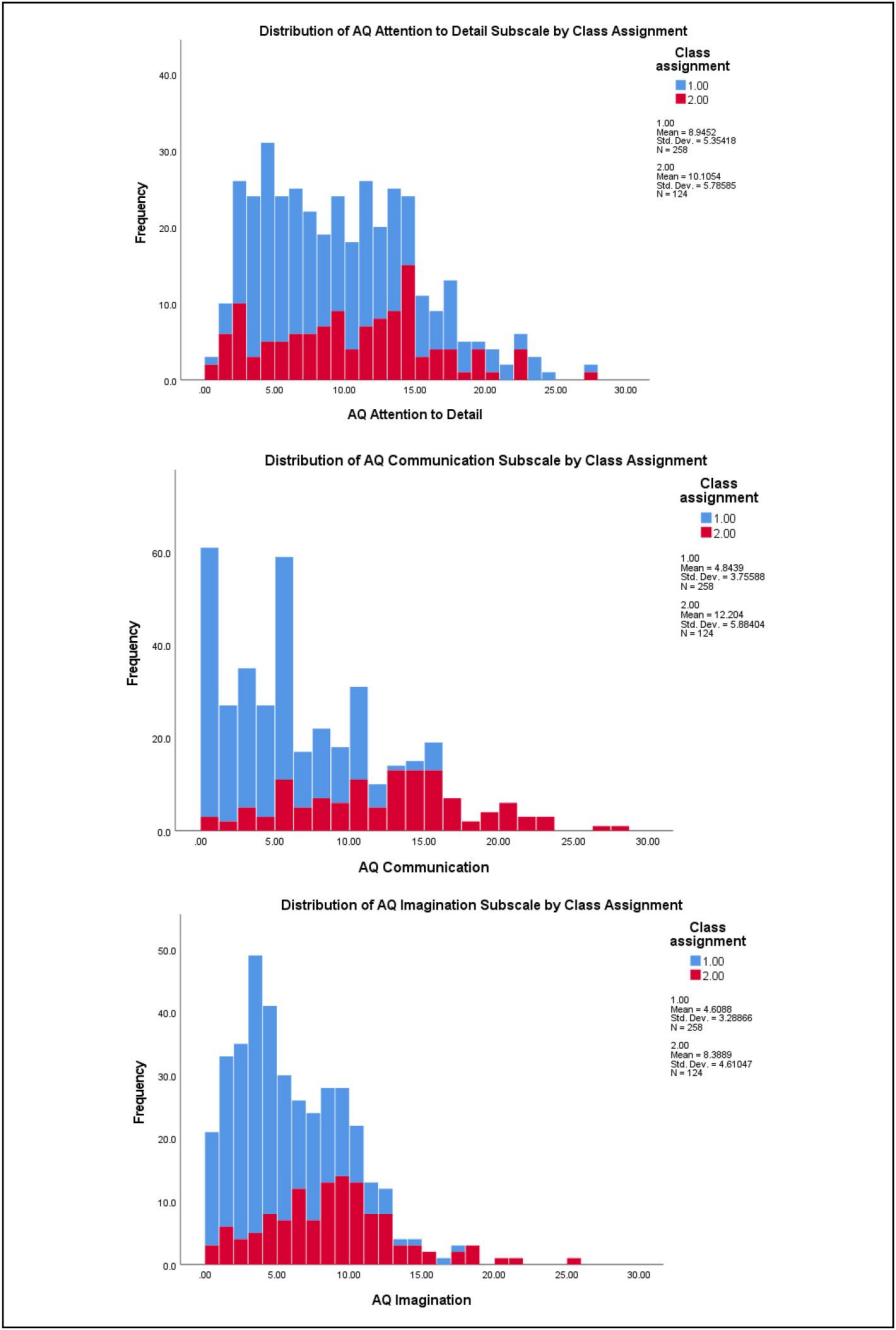

FIGURE 4Replication sample: Histogram of CPRS subscale score frequencies by factor mixture modelling class assignment.

**Figure:**
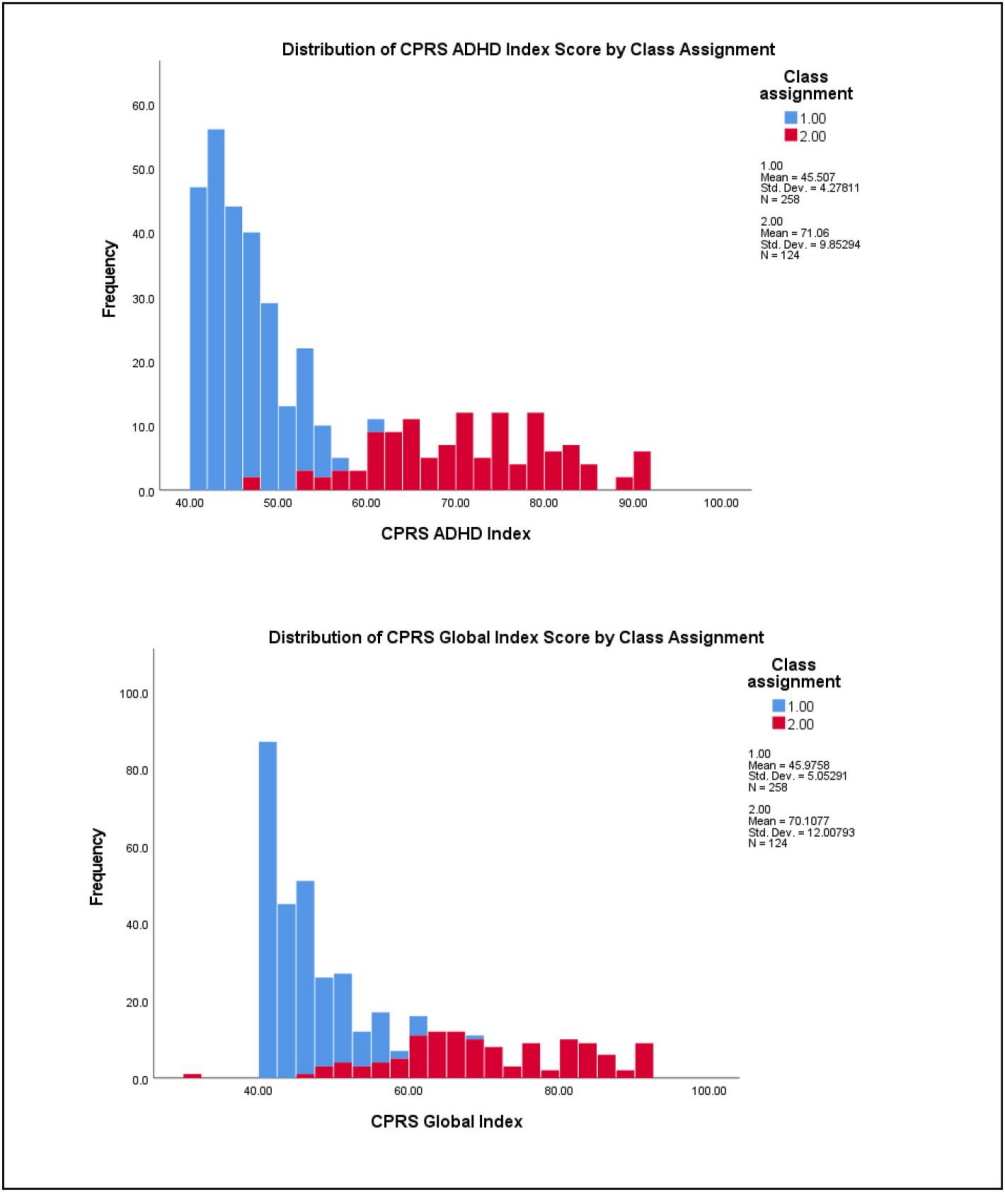

**Figure:**
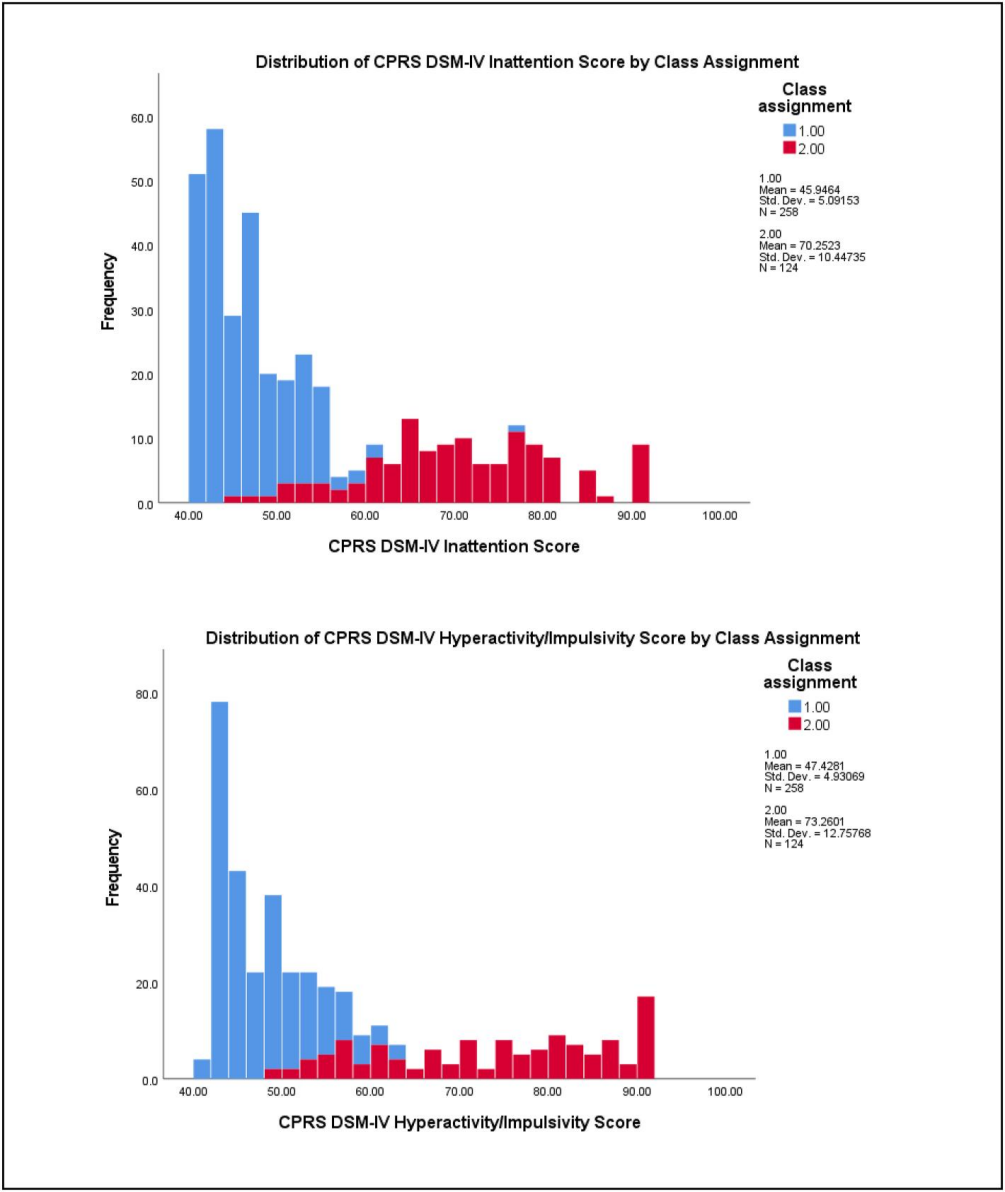

## DISCUSSION

The common co‐occurrence of ADHD and autistic traits is increasingly recognised in research and clinical practice (Antshel & Russo, 2019). Although previous studies have described elevated levels of autistic traits in children diagnosed with ADHD compared to their neurotypical peers, no studies have established a baseline distribution of subclinical autistic traits in children with ADHD. Our study presents a factor mixture model of the distribution of parent‐reported subclinical autistic and ADHD traits in Australian children aged 6–15 years with and without an ADHD diagnosis. The findings demonstrate that subclinical autistic traits were unevenly distributed in children with clinically significant levels of ADHD traits in two independent samples. Our findings also highlight the potential clinical utility of assessing subclinical autistic traits among children with ADHD, particularly in relation to social communication. Additionally, our results indicate that both dimensional and categorical conceptualisations of neurodevelopmental traits have merit.

We found that ADHD and autistic traits formed two distinct factors as hypothesised. Next, and consistent with existing literature (e.g., Deserno et al., 2022; Posner et al., 2020), the distribution of CPRS ratings in both our samples supported the description of ADHD as a continuum extending from neurotypical profiles. We were also able to successfully replicate a 2‐factor, 2‐class FMM‐3 model in two independent samples based on responses to the AQ and CPRS. This model captured two classes of participants that significantly differed along the latent factors of ‘autism’ and ‘ADHD’, as estimated by the AQ and CPRS respectively. Each class mostly consisted of either neurotypical children or children diagnosed with ADHD. This indicates that the current categorical diagnostic system had utility in identifying key differences between neurotypical compared with neurodivergent presentations in these samples.

There was more variability in the distribution of subclinical autistic traits between the two classes across the samples. At the subscale level, participants in Class 2 demonstrated significantly higher mean ratings on measures of ADHD and autistic traits than those in Class 1. This indicates that most children who exhibit clinically significant levels of ADHD traits were also rated as demonstrating higher levels of autistic traits than children with low levels of ADHD traits (i.e., likely neurotypical) in these two samples. Visual inspection of their distribution (see Figures 1–4) indicated that this elevation was uneven across varying levels of ADHD traits. This tentatively suggests that there may be a combination of shared and independent aetiological drivers of subclinical autistic traits in children with clinically significant ADHD traits, consistent with recent genetics findings (Mattheisen et al., 2022). However, further modelling of these traits in a sample that also includes children who have co‐occurring autism and ADHD would need to be undertaken in order to explore this in more detail.

The largest class effect on the AQ was detected for the Communication subscale across both samples. The AQ Communication subscale encompasses aspects of pragmatic language and social inference, which are known to be weaker in children with ADHD compared to their neurotypical peers (Kessler & Ikuta, 2023). There are some suggestions from previous studies that these weaknesses may not be inherent to ADHD. First, similarities between the social communication profile of children with ADHD to those of autistic children have been identified, and were shown to be reflective of more than just the child's internalising, externalising, and ADHD traits (Grzadzinski et al., 2011). Similar patterns of association between regional brain volumes and social cognitive measures in autism and ADHD have also been detected (Baribeau et al., 2019). Finally, previous studies of neurotypical children have only demonstrated a partial mediation of social communicative skills by hyperactive/impulsive traits (Rints et al., 2015). As such, it is possible that there are other factors underpinning difficulties in the application of pragmatic rules in social interactions beyond a child's hyperactivity and/or impulsivity, and that these could resemble, or be attributable to, traits on the autism spectrum. Where these pragmatic language challenges are identified clinically in children with ADHD, and regardless of their aetiology (i.e., autism or otherwise), it is worthwhile considering a referral to a speech and language therapist for further support.

Taken together, the results suggest that screening for subclinical autistic traits in children with ADHD may be clinically meaningful, particularly if difficulties with social communication are described. Given that subclinical autistic traits can index cognitive and clinical challenges in children with ADHD (Cooper et al., 2014), there may be important implications for intervention if these traits are identified. Finally, if it is indeed the case that social communication challenges are inherent to ADHD and sufficiently distinct from similar challenges described in autistic populations, then there may be additional implications for the discriminant validity of social communication scales in ADHD populations.

### Strengths and limitations

Our study has two key strengths. To date, the debate between categorical and dimensional conceptualisations of these neurotypes remains ongoing and the use of FMM is a helpful addition to the adjudication of this debate because it does not “force” (Clark et al., 2013) either a categorical or dimensional structure to the data, but it instead allows for the underlying heterogeneity to be modelled in *both* ways. Our findings provide an example of where dimensional models of psychological differences (e.g., Research Domain Criteria [RDoC]; Insel et al., 2010) can be unified with existing categorical nosologies to capture aspects of within‐group and between‐group trait heterogeneity. Second, the successful replication of the 2‐factor, 2‐class model across two distinct, independent samples confers greater confidence in our results (Bauer & Curran, 2004).

Future studies could build on our findings by addressing some of our limitations. The exclusion of children with commonly co‐occurring neurodevelopmental diagnoses, such as a specific learning disorder or intellectual developmental disability may have biased our analyses towards a particular model of autistic and ADHD traits. Including these children may reduce this potential bias and allow future studies to investigate whether our model is replicable in a more phenotypically heterogeneous sample of children with ADHD.

Additionally, our study only used parent‐reported measures of observable behaviours, which have limitations (Gianarris et al., 2001; Gomez et al., 2019). As such, children's subjective experiences could not be described. This may have missed vital and core features of their presentation, such as inattentive traits (particularly in females); (Hinshaw et al., 2022), their sensory preferences and masking tendencies, in addition to ADHD traits obscuring autistic traits (i.e., diagnostic overshadowing; Miodovnik et al., 2015; Kentrou et al., 2019). Moreover, our analyses were collapsed across a wide age range which restricted the ability of our models to capture developmental changes in autistic and ADHD trait presentation (Hartman et al., 2016; Shakeshaft et al., 2023; Visser et al., 2016). As environmental demands evolve, previously unrecognised autistic traits may become more recognisable (Davidovitch, Levit‐Binnun & Manning‐Courtney, 2015). These two limitations may have contributed to the potential confounding effect of age in our results where older children were more likely to be allocated to the neurotypical class. It is also possible that these differences reflect the operation of an inadvertent selection bias in our sample recruitment. The validity of our models could, in future, be evaluated through the application of FMM to different parent‐ and child‐report measures of autistic and ADHD traits from larger, representative populations that are stratified by age and sex. Studies may also consider including multiple measures of autistic and ADHD traits to strengthen the empirical support provided by latent variable modelling analyses for the existence of latent ‘autism’ and ‘ADHD’ constructs. Finally, some participating children may have also been autistic, but were undiagnosed, thereby inflating AQ and CPRS scores. However, we expect that this would not have changed our models significantly given that a wide distribution of AQ and CPRS scores were observed across both samples.

## CONCLUSION

We demonstrated in two independent samples that most children with ADHD also exhibited elevated levels of subclinical autistic traits compared to neurotypical peers. The findings support conceptualising ADHD as continuously distinct from neurotypical presentations, while highlighting the utility of current diagnostic criteria to distinguish between neurotypical and neurodivergent profiles. There may be clinical utility in screening for subclinical autistic traits in children with ADHD and in monitoring for changes in their presentation across development.

## AUTHOR CONTRIBUTIONS

**Tracey Chau**: Conceptualization; formal analysis; methodology; writing – original draft; writing – review & editing. **Jeggan Tiego**: Conceptualization; formal analysis; supervision; writing – review & editing. **Louise E. Brown**: Validation; writing – review & editing. **Olivia J. Mellahn**: Methodology; project administration; validation; writing – review & editing. **Beth P. Johnson**: Methodology; project administration; supervision; writing – review & editing. **Aurina Arnatkeviciute**: Data curation; writing – review & editing. **Ben D. Fulcher**: Data curation; writing – review & editing. **Natasha Matthews**: Data curation; investigation; methodology; project administration; writing – review & editing. **Mark A. Bellgrove**: Conceptualization; data curation; funding acquisition; investigation; project administration; resources; supervision; writing – review & editing.

## CONFLICT OF INTEREST STATEMENT

The authors declare no conflicts of interest.

## ETHICAL CONSIDERATIONS

Written consent to participate was obtained from parents, while verbal assent was obtained from children. The MAGNET project was approved by Monash University Human Research Ethics Committee (CF16/1537–2016000806), Department of Education and Training Victoria Human Research Ethics Committee (2017_003570), and Monash Health Human Research Ethics Committee (RES‐19‐0000‐372A). The Biomarkers study was approved by the Royal Children's Hospital and The University of Queensland Human Research Ethics Committees.

## Supporting information

Supporting Information S1

## Data Availability

The data that support the findings of this study are available from the corresponding author upon reasonable request.
