# Supplementary material for: The distribution of parent‐reported attention‐deficit/hyperactivity disorder and subclinical autistic traits in children with and without an ADHD diagnosis
Source: JCPP Adv. 2024 Feb 24;4(2):e12223. doi: 10.1002/jcv2.12223 (PMC11143953; doi:10.1002/jcv2.12223)
Supplement: Supplementary file 1 — Supporting Information S1 [file JCV2-4-e12223-s001.docx]

**Supplementary Materials**

Appendix S1.

*Discriminant Validity*

We used two approaches to test the discriminant validity of the ADHD and autistic trait factors (i.e., to determine they were empirically distinct dimensions; Hair, 2014; Fornell and Larcker, 1981). First, we examined the 95% confidence interval for the factor intercorrelation. If the interval did not contain 1.00, then this suggests that the factors are empirically distinct. Second, we calculated and compared the average variance extracted (i.e., the average of the sum of variance explained by the factor in its indicators) with the squared correlation (i.e., the squared factor intercorrelation, which indicates how much variance is shared between the factors). If the average variance extracted was greater than the squared correlation, then this provides further evidence of discriminant validity.

Appendix S2.

*Model Selection*

This section was written based on Nylund et al., 2007’s recommendations. Model selection involves evaluating the Bayesian Information Criterion (BIC; lower is better) and Vuong-Lo-Mendell-Rubin adjusted likelihood ratio test *p* values (VLMR*p*; Lo et al., 2001) of competing models and, where available, using the parametric bootstrapped likelihood ratio test *p* value (BLRT*p*; McLachlan, 1987) to adjudicate in cases where the BIC and VLMR*p* do not reach a consensus. Best model fit is also determined based on substantive interpretation and on a balance of other commonly used fit statistics, such as: the log likelihood (lower is better), entropy value (values approaching 1 indicate good class separation; Greenbaum et al., 2005) and the Akaike Information Criterion (AIC; lower is better). Bayes factors (BF; Jeffreys, 1961) were also calculated to quantify the relative strength of fit of the chosen model in relation to the CFA model based on recommended interpretations (Andraszewicz et al., 2015).

Table S1.

*Discovery Sample: Descriptive Statistics for Continuous Variables*

|  | N | Minimum | Maximum | Mean | Std. Deviation |
| --- | --- | --- | --- | --- | --- |
| Age | 208 | 4 | 15 | 8.21 | 2.77 |
| FSIQ | 197 | 76 | 141 | 105.08 | 13.06 |
| AQ_Soc | 203 | 0 | 26 | 8.86 | 6.18 |
| AQ_Att_Sw | 203 | 0 | 27 | 12.76 | 6.64 |
| AQ_Att_Det | 203 | 1 | 29 | 12.94 | 5.86 |
| AQ_Com | 203 | 0 | 27 | 11.19 | 6.55 |
| AQ_Imag | 203 | 0 | 22 | 8.08 | 4.98 |
| CADHD | 204 | 40 | 90 | 60.95 | 14.68 |
| CCGI | 204 | 38 | 90 | 60.84 | 15.67 |
| CDSM_Inatt | 204 | 40 | 90 | 60.50 | 14.74 |
| CDSM_Hyp | 204 | 40 | 90 | 61.25 | 15.36 |

*Note.* FSIQ = Full scale IQ; AQ_Soc = AQ Social Skill subscale; AQ_Att_Sw = AQ Attention Switching subscale; AQ_Att_Det = AQ Attention to Detail subscale; AQ_Com = AQ Communication subscale; AQ_Imag = AQ Imagination subscale; CADHD = CPRS ADHD Index; CCGI = CPRS Global Index; CDSM_Inatt = CPRS DSM-IV Inattention score; CDSM_Hyp = CPRS DSM-IV Hyperactivity/Impulsivity score; Conners_DSM_Tot = CPRS DSM-IV Total score

Table S2.

*Discovery Sample: Shapiro-Wilk (W) Test for Normality Results*

|  | *W* Statistic | df | Sig. |
| --- | --- | --- | --- |
| Age | .951 | 192 | < .001 |
| FSIQ | .992 | 192 | .423 |
| AQ_Soc | .952 | 192 | < .001 |
| AQ_Att_Sw | .977 | 192 | .003 |
| AQ_Att_Det | .966 | 192 | < .001 |
| AQ_Com | .975 | 192 | .001 |
| AQ_Imag | .960 | 192 | < .001 |
| CADHD | .945 | 192 | < .001 |
| CCGI | .934 | 192 | < .001 |
| CDSM_Inatt | .941 | 192 | < .001 |
| CDSM_Hyp | .913 | 192 | < .001 |

*Note.* FSIQ = Full scale IQ; AQ_Soc = AQ Social Skill subscale; AQ_Att_Sw = AQ Attention Switching subscale; AQ_Att_Det = AQ Attention to Detail subscale; AQ_Com = AQ Communication subscale; AQ_Imag = AQ Imagination subscale; CADHD = CPRS ADHD Index; CCGI = CPRS Global Index; CDSM_Inatt = CPRS DSM-IV Inattention score; CDSM_Hyp = CPRS DSM-IV Hyperactivity/Impulsivity score.

Figure S1.

*Discovery Sample: Confirmatory Factor Analysis Diagram (Original Model)*


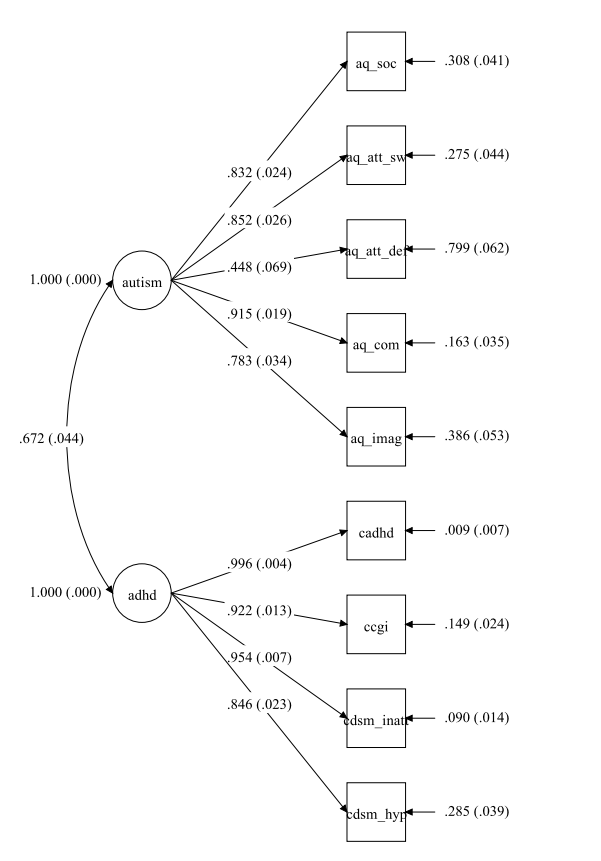


*Note.* Standardised estimates are shown with standard errors in parentheses; AQ_Soc = AQ Social Skill subscale; AQ_Att_Sw = AQ Attention Switching subscale; AQ_Att_Det = AQ Attention to Detail subscale; AQ_Com = AQ Communication subscale; AQ_Imag = AQ Imagination subscale; CADHD = CPRS ADHD Index; CCGI = CPRS Global Index; CDSM_Inatt = CPRS DSM-IV Inattention score; CDSM_Hyp = CPRS DSM-IV Hyperactivity/Impulsivity score.

Figure S2.

*Discovery Sample: Confirmatory Factor Analysis Diagram (Modified Model)*


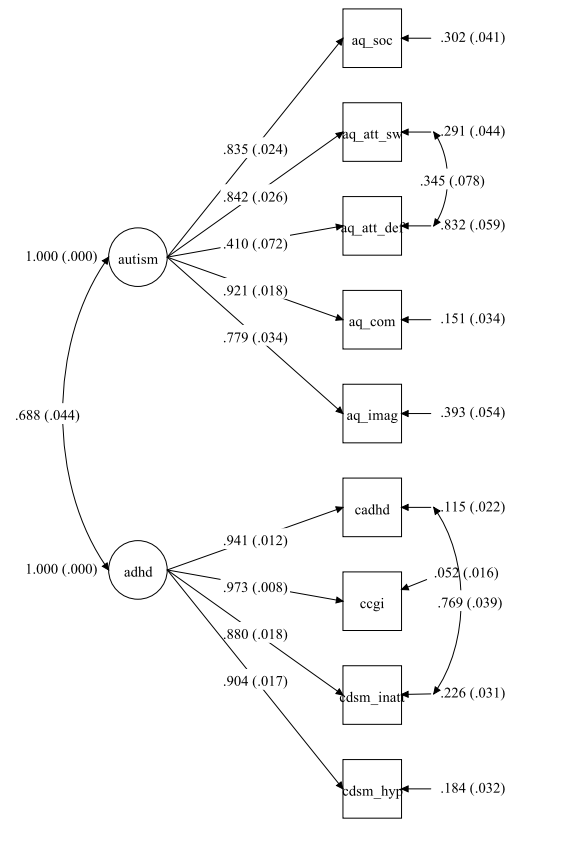


*Note.* Standardised estimates are shown with standard errors in parentheses; AQ_Soc = AQ Social Skill subscale; AQ_Att_Sw = AQ Attention Switching subscale; AQ_Att_Det = AQ Attention to Detail subscale; AQ_Com = AQ Communication subscale; AQ_Imag = AQ Imagination subscale; CADHD = CPRS ADHD Index; CCGI = CPRS Global Index; CDSM_Inatt = CPRS DSM-IV Inattention score; CDSM_Hyp = CPRS DSM-IV Hyperactivity/Impulsivity score.

Table S3.

*Discovery Sample: Confirmatory Factor Analysis Correlation Residuals – Original Model*

|  | AQ_Soc | AQ_Att_Sw | AQ_Att_Det | AQ_Com | AQ_Imag | CADHDTScore | CCGI | CDSM_Inatt | CDSM_Hyp |
| --- | --- | --- | --- | --- | --- | --- | --- | --- | --- |
| AQ_Soc | .000 |  |  |  |  |  |  |  |  |
| AQ_Att_Sw | .005 | .000 |  |  |  |  |  |  |  |
| AQ_Att_Det | - .071 | **.133** | .000 |  |  |  |  |  |  |
| AQ_Com | .009 | - .020 | - .039 | .000 |  |  |  |  |  |
| AQ_Imag | .015 | - .015 | .038 | .012 | .000 |  |  |  |  |
| CADHD | - .051 | .043 | - .066 | .024 | - .072 | .000 |  |  |  |
| CCGI | - .021 | .088 | .011 | .048 | - .047 | - .001 | .000 |  |  |
| CDSM_Inatt | - .053 | .023 | **- .102** | .048 | - .037 | .002 | - .020 | .000 |  |
| CDSM_Hyp | - .017 | **.131** | .058 | **.121** | .009 | - .003 | **.101** | - .036 | .000 |

*Note.* **Bolded** = exceeds recommended cut-off of |.10| (Kline, 2015); FSIQ = Full scale IQ; AQ_Soc = AQ Social Skill subscale; AQ_Att_Sw = AQ Attention Switching subscale; AQ_Att_Det = AQ Attention to Detail subscale; AQ_Com = AQ Communication subscale; AQ_Imag = AQ Imagination subscale; CADHD = CPRS ADHD Index; CCGI = CPRS Global Index; CDSM_Inatt = CPRS DSM-IV Inattention score; CDSM_Hyp = CPRS DSM-IV Hyperactivity/Impulsivity score

Table S4.

*Discovery Sample: Confirmatory Factor Analysis Correlation Residuals – Modified Model*

|  | AQ_Soc | AQ_Att_Sw | AQ_Att_Det | AQ_Com | AQ_Imag | CADHDTScore | CCGI | CDSM_Inatt | CDSM_Hyp |
| --- | --- | --- | --- | --- | --- | --- | --- | --- | --- |
| AQ_Soc | .000 |  |  |  |  |  |  |  |  |
| AQ_Att_Sw | .010 | .000 |  |  |  |  |  |  |  |
| AQ_Att_Det | - .041 | .000 | .000 |  |  |  |  |  |  |
| AQ_Com | .001 | - .017 | - .006 | .000 |  |  |  |  |  |
| AQ_Imag | .016 | - .005 | .070 | .011 | .000 |  |  |  |  |
| CADHD | - .035 | .068 | - .032 | .040 | - .053 | .000 |  |  |  |
| CCGI | - .065 | .051 | .014 | - .002 | - .084 | .002 | .000 |  |  |
| CDSM_Inatt | - .026 | .059 | - .063 | .077 | - .007 | .000 | .004 | .000 |  |
| CDSM_Hyp | - .064 | .092 | .057 | .068 | - .030 | - .011 | .002 | - .024 | .000 |

*Note.* FSIQ = Full scale IQ; AQ_Soc = AQ Social Skill subscale; AQ_Att_Sw = AQ Attention Switching subscale; AQ_Att_Det = AQ Attention to Detail subscale; AQ_Com = AQ Communication subscale; AQ_Imag = AQ Imagination subscale; CADHD = CPRS ADHD Index; CCGI = CPRS Global Index; CDSM_Inatt = CPRS DSM-IV Inattention score; CDSM_Hyp = CPRS DSM-IV Hyperactivity/Impulsivity score

Table S5.

*Discovery Sample: Confirmatory Factor Analysis Regression Coefficients*

|  | Original model | | Modified model | |
| --- | --- | --- | --- | --- |
|  | Estimate (S.E.) | Standardised estimate (S.E.) | Estimate (S.E.) | Standardised estimate (S.E.) |
| *ADHD* |  |  |  |  |
| CADHD | 1.000 (.000) | .996 (.004) | 1.000 (.000) | .941 (.012) |
| CCGI | .989 (.031) | .922 (.013) | 1.104 (.031) | .973 (.008) |
| CDSM_Inatt | .962 (.018) | .954 (.007) | .939 (.019) | .880 (.018) |
| CDSM_Hyp | .889 (.037) | .846 (.023) | 1.005 (.041) | .904 (.017) |
| *Autism* |  |  |  |  |
| AQ_Soc | 1.000 (.000) | .832 (.024) | 1.000 (.000) | .835 (.024) |
| AQ_Att_Sw | 1.100 (.075) | .852 (.026) | 1.083 (.074) | .842 (.026) |
| AQ_Att_Det | .511 (.088) | .448 (.069) | .465 (.088) | .410 (.072) |
| AQ_Com | 1.165 (.064) | .915 (.019) | 1.168 (.066) | .921 (.018) |
| AQ_Imag | .759 (.055) | .783 (.034) | .752 (.055) | .779 (.034) |
| ADHD with Autism | 50.194 (5.363) | .672 (.044) | 48.779 (5.431) | .688 (.044) |

*Note*. All regression coefficients were significant (*p* < .001); S.E. = standard error; AQ_Soc = AQ Social Skill subscale; AQ_Att_Sw = AQ Attention Switching subscale; AQ_Att_Det = AQ Attention to Detail subscale; AQ_Com = AQ Communication subscale; AQ_Imag = AQ Imagination subscale; CADHD = CPRS ADHD Index; CCGI = CPRS Global Index; CDSM_Inatt = CPRS DSM-IV Inattention score; CDSM_Hyp = CPRS DSM-IV Hyperactivity/Impulsivity score

Table S6.

*Discovery Sample: 2 factor, 2 class FMM-3 – BCH Procedure Chi-Square Values*

| Auxiliary variable | Chi-square value | *p* value |
| --- | --- | --- |
| Age | 4.23 | .040 |
| FSIQ | 4.66 | .031 |

Table S7.

*Discovery Sample: 2 factor, 2 class FMM-3 – BCH Procedure Means and Standard Errors*

| Variable | Mean | Standard Error |
| --- | --- | --- |
| *Age* |  |  |
| Class 1 | 8.48 | .289 |
| Class 2 | 7.62 | .297 |
| *FSIQ* |  |  |
| Class 1 | 107.03 | 1.26 |
| Class 2 | 102.70 | 1.64 |

Table S8.

*Discovery Sample: MANOVA Results – AQ and CPRS Subscales by Factor Mixture Modelling Class Assignment*

| Subscale | *F* value | Effect size (partial *η*^2^) |
| --- | --- | --- |
| *AQ* |  |  |
| AQ_Soc | *F*(1, 201) = 24.63** | .109 |
| AQ_Att_Sw | *F*(1, 201) = 73.16** | .267 |
| AQ_Att_Det | *F**(1, 128.742) = 23.305** | .198 |
| AQ_Com | *F*(1, 201) = 81.23** | .288 |
| AQ_Imag | *F*(1, 201) = 26.79** | .118 |
| *CPRS* |  |  |
| CADHD | *F*(1, 201) = 144.22** | .418 |
| CCGI | *F*(1, 201) = 253.569** | .558 |
| CDSM_Inatt | *F*(1, 201) = 93.25** | .317 |
| CDSM_Hyp | *F*(1, 201) = 606.18** | .751 |

*Note*. ** = Significant at *p* <.001; ­*F** = Brown-Forsythe *F*-statistic; AQ_Soc = AQ Social Skill subscale; AQ_Att_Sw = AQ Attention Switching subscale; AQ_Att_Det = AQ Attention to Detail subscale; AQ_Com = AQ Communication subscale; AQ_Imag = AQ Imagination subscale; CADHD = CPRS ADHD Index; CCGI = CPRS Global Index; CDSM_Inatt = CPRS DSM-IV Inattention score; CDSM_Hyp = CPRS DSM-IV Hyperactivity/Impulsivity score

Table S9.

*Replication Sample: Descriptive Statistics for Continuous Variables (Post-Imputation)*

|  | N | Minimum | Maximum | Mean | Std. Deviation |
| --- | --- | --- | --- | --- | --- |
| Age | 382 | 6 | 15 | 10.93 | 2.15 |
| FSIQ | 382 | 74 | 141 | 104.61 | 13.04 |
| AQ_Soc | 382 | 0 | 30 | 6.16 | 5.05 |
| AQ_Att_Sw | 382 | 0 | 27 | 8.61 | 5.57 |
| AQ_Att_Det | 382 | 0 | 27 | 9.32 | 5.52 |
| AQ_Com | 382 | 0 | 28 | 7.23 | 5.71 |
| AQ_Imag | 382 | 0 | 25 | 5.84 | 4.16 |
| CADHD | 382 | 40 | 90 | 53.80 | 13.68 |
| CCGI | 382 | 30 | 90 | 53.81 | 13.85 |
| CDSM_Inatt | 382 | 40 | 90 | 53.84 | 13.51 |
| CDSM_Hyp | 382 | 41 | 90 | 55.81 | 14.68 |

*Note.* FSIQ = Full scale IQ; AQ_Soc = AQ Social Skill subscale; AQ_Att_Sw = AQ Attention Switching subscale; AQ_Att_Det = AQ Attention to Detail subscale; AQ_Com = AQ Communication subscale; AQ_Imag = AQ Imagination subscale; CADHD = CPRS ADHD Index; CCGI = CPRS Global Index; CDSM_Inatt = CPRS DSM-IV Inattention score; CDSM_Hyp = CPRS DSM-IV Hyperactivity/Impulsivity score.

Table S10.

*Replication Sample: Shapiro-Wilk (W) Test for Normality Results (Post-Imputation)*

|  | *W* Statistic | df | Sig. |
| --- | --- | --- | --- |
| Age | .956 | 382 | < .001 |
| FSIQ | .991 | 382 | .024 |
| AQ_Soc | .915 | 382 | < .001 |
| AQ_Att_Sw | .961 | 382 | < .001 |
| AQ_Att_Det | .964 | 382 | < .001 |
| AQ_Com | .927 | 382 | < .001 |
| AQ_Imag | .936 | 382 | < .001 |
| CADHD | .842 | 382 | < .001 |
| CCGI | .839 | 382 | < .001 |
| CDSM_Inatt | .855 | 382 | < .001 |
| CDSM_Hyp | .810 | 382 | < .001 |

*Note.* FSIQ = Full scale IQ; AQ_Soc = AQ Social Skill subscale; AQ_Att_Sw = AQ Attention Switching subscale; AQ_Att_Det = AQ Attention to Detail subscale; AQ_Com = AQ Communication subscale; AQ_Imag = AQ Imagination subscale; CADHD = CPRS ADHD Index; CCGI = CPRS Global Index; CDSM_Inatt = CPRS DSM-IV Inattention score; CDSM_Hyp = CPRS DSM-IV Hyperactivity/Impulsivity score.

Figure S3.

*Replication Sample: Confirmatory Factor Analysis Diagram (Original Model)*

*
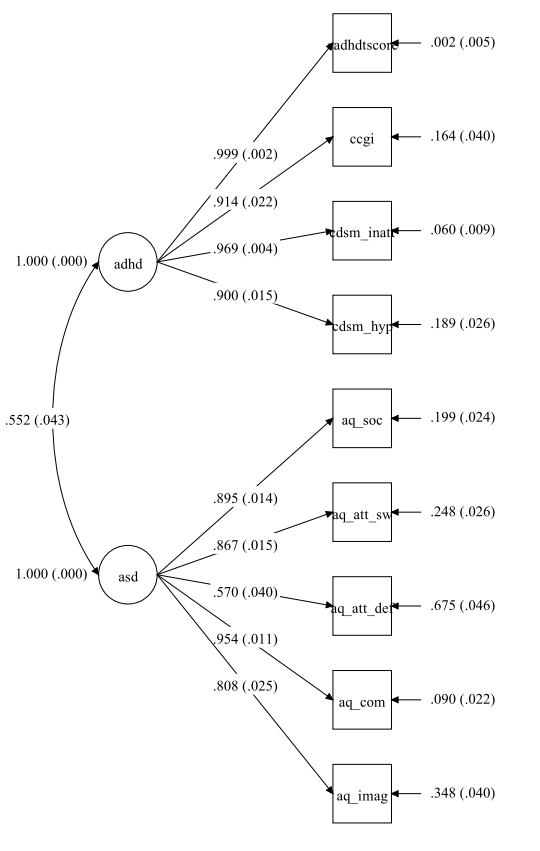
*

*Note.* Standardised estimates are shown with standard errors in parentheses; AQ_Soc = AQ Social Skill subscale; AQ_Att_Sw = AQ Attention Switching subscale; AQ_Att_Det = AQ Attention to Detail subscale; AQ_Com = AQ Communication subscale; AQ_Imag = AQ Imagination subscale; CADHD = CPRS ADHD Index; CCGI = CPRS Global Index; CDSM_Inatt = CPRS DSM-IV Inattention score; CDSM_Hyp = CPRS DSM-IV Hyperactivity/Impulsivity score.

Figure S4.

*Replication Sample: Confirmatory Factor Analysis Diagram (Modified Model)*


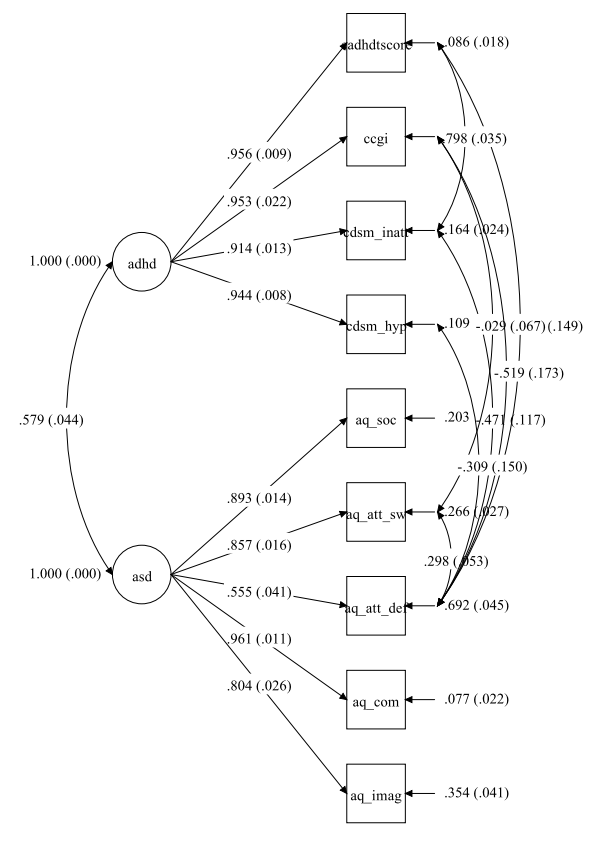


*Note.* Standardised estimates are shown with standard errors in parentheses; AQ_Soc = AQ Social Skill subscale; AQ_Att_Sw = AQ Attention Switching subscale; AQ_Att_Det = AQ Attention to Detail subscale; AQ_Com = AQ Communication subscale; AQ_Imag = AQ Imagination subscale; CADHD = CPRS ADHD Index; CCGI = CPRS Global Index; CDSM_Inatt = CPRS DSM-IV Inattention score; CDSM_Hyp = CPRS DSM-IV Hyperactivity/Impulsivity score.

Table S11.

*Replication Sample: Confirmatory Factor Analysis Correlation Residuals – Original Model*

|  | AQ_Soc | AQ_Att_Sw | AQ_Att_Det | AQ_Com | AQ_Imag | CADHDTScore | CCGI | CDSM_Inatt | CDSM_Hyp |
| --- | --- | --- | --- | --- | --- | --- | --- | --- | --- |
| AQ_Soc | .000 |  |  |  |  |  |  |  |  |
| AQ_Att_Sw | .005 | .000 |  |  |  |  |  |  |  |
| AQ_Att_Det | - .035 | **.120** | .000 |  |  |  |  |  |  |
| AQ_Com | .001 | - .005 | - .023 | .000 |  |  |  |  |  |
| AQ_Imag | .007 | - .004 | .061 | - .005 | .000 |  |  |  |  |
| CADHDTScore | - .017 | - .084 | **- .198** | .054 | - .019 | .000 |  |  |  |
| CCGI | - .008 | - .069 | **- .168** | .063 | - .008 | .000 | .000 |  |  |
| CDSM_Inatt | - .022 | - .081 | **- .206** | .045 | - .032 | .000 | - .008 | .000 |  |
| CDSM_Hyp | .000 | - .039 | - .099 | **.109** | .022 | .000 | .078 | - .020 | .000 |

*Note.* **Bolded** = exceeds recommended cut-off of |.10| (Kline, 2015); AQ_Soc = AQ Social Skill subscale; AQ_Att_Sw = AQ Attention Switching subscale; AQ_Att_Det = AQ Attention to Detail subscale; AQ_Com = AQ Communication subscale; AQ_Imag = AQ Imagination subscale; CADHD = CPRS ADHD Index; CCGI = CPRS Global Index; CDSM_Inatt = CPRS DSM-IV Inattention score; CDSM_Hyp = CPRS DSM-IV Hyperactivity/Impulsivity score.

Table S12.

*Replication Sample: Confirmatory Factor Analysis Correlation Residuals – Modified Model*

|  | AQ_Soc | AQ_Att_Sw | AQ_Att_Det | AQ_Com | AQ_Imag | CADHDTScore | CCGI | CDSM_Inatt | CDSM_Hyp |
| --- | --- | --- | --- | --- | --- | --- | --- | --- | --- |
| AQ_Soc | .000 |  |  |  |  |  |  |  |  |
| AQ_Att_Sw | .016 | .000 |  |  |  |  |  |  |  |
| AQ_Att_Det | - .020 | .012 | .000 |  |  |  |  |  |  |
| AQ_Com | - .030 | - .001 | - .012 | .000 |  |  |  |  |  |
| AQ_Imag | .012 | .008 | .076 | - .007 | .000 |  |  |  |  |
| CADHDTScore | - .018 | - .080 | - .038 | .048 | - .019 | .000 |  |  |  |
| CCGI | - .049 | - .100 | - .055 | .014 | - .044 | .002 | .000 |  |  |
| CDSM_Inatt | - .016 | - .070 | - .036 | .046 | - .025 | .000 | .007 | .000 |  |
| CDSM_Hyp | - .044 | - .077 | - .034 | .057 | - .016 | - .003 | .001 | - .010 | .000 |

*Note.* AQ_Soc = AQ Social Skill subscale; AQ_Att_Sw = AQ Attention Switching subscale; AQ_Att_Det = AQ Attention to Detail subscale; AQ_Com = AQ Communication subscale; AQ_Imag = AQ Imagination subscale; CADHD = CPRS ADHD Index; CCGI = CPRS Global Index; CDSM_Inatt = CPRS DSM-IV Inattention score; CDSM_Hyp = CPRS DSM-IV Hyperactivity/Impulsivity score.

Table S13.

*Replication Sample: Confirmatory Factor Analysis Regression Coefficients*

|  | Original model | | Modified model | |
| --- | --- | --- | --- | --- |
|  | Estimate (S.E.) | Standardised estimate (S.E.) | Estimate (S.E.) | Standardised estimate (S.E.) |
| *ADHD* |  |  |  |  |
| CADHD | 1.000 (.000) | .999 (.002) | 1.000 (.000) | .956 (.009) |
| CCGI | .926 (.034) | .914 (.022) | 1.008 (.035) | .953 (.022) |
| CDSM_Inatt | .958 (.014) | .969 (.004) | .944 (.015) | .914 (.013) |
| CDSM_Hyp | .967 (.032) | .900 (.015) | 1.060 (.029) | .944 (.008) |
| *Autism* |  |  |  |  |
| AQ_Social | 1.000 (.000) | .895 (.014) | 1.000 (.000) | .893 (.014) |
| AQ_Att_Sw | 1.067 (.055) | .867 (.015) | 1.056 (.055) | .857 (.016) |
| AQ_Att_Det | .695 (.062) | .570 (.040) | .671 (.060) | .555 (.041) |
| AQ_Com | 1.204 (.041) | .954 (.011) | 1.216 (.042) | .961 (.011) |
| AQ_Imag | .742 (.036) | .808 (.025) | .741 (.036) | .804 (.026) |
| ADHD with Autism | 34.050 (4.065) | .552 (.043) | 34.102 (4.019) | .579 (.044) |

*Note*. All regression coefficients were significant (*p* < .001); S.E. = standard error.

Table S14.

*Replication Sample: 2 factor, 2 class FMM-3 – BCH Procedure Chi-Square Values*

| Auxiliary variable | Chi-square value | *p* value |
| --- | --- | --- |
| Age | 18.34 | < .001 |
| FSIQ | 60.87 | < .001 |

Table S15.

*Replication Sample: 2 factor, 2 class FMM-3 – BCH Procedure Means and Standard Errors*

| Variable | Mean | Standard Error |
| --- | --- | --- |
| *Age* |  |  |
| Class 1 | 11.27 | .14 |
| Class 2 | 10.25 | .19 |
| *FSIQ* |  |  |
| Class 1 | 108.29 | .756 |
| Class 2 | 97.24 | 1.16 |

Table S16.

*Replication Sample: MANOVA Results – AQ and CPRS Subscales by Factor Mixture Modelling Class Assignment*

| Subscale | *F* value | Effect size (partial *η*^2^) |
| --- | --- | --- |
| *AQ* |  |  |
| AQ_Soc | *F*(1, 380) = 136.69** | .265 |
| AQ_Att_Sw | *F*(1, 380) = 68.50** | .153 |
| AQ_Att_Det | *F*(1, 380) = 3.73 | .010 |
| AQ_Com | *F*(1, 380) = 218.67** | .365 |
| AQ_Imag | *F*(1, 380) = 84.30** | .182 |
| *CPRS* |  |  |
| CADHD | *F*(1, 380) = 1248.45** | .767 |
| CCGI | *F*(1, 380) = 762.76** | .667 |
| CDSM_Inatt | *F*(1, 380) = 935.96** | .711 |
| CDSM_Hyp | *F*(1, 380) = 808.46** | .680 |

*Note*. ** = Significant at *p* <.001; ­AQ_Soc = AQ Social Skill subscale; AQ_Att_Sw = AQ Attention Switching subscale; AQ_Att_Det = AQ Attention to Detail subscale; AQ_Com = AQ Communication subscale; AQ_Imag = AQ Imagination subscale; CADHD = CPRS ADHD Index; CCGI = CPRS Global Index; CDSM_Inatt = CPRS DSM-IV Inattention score; CDSM_Hyp = CPRS DSM-IV Hyperactivity/Impulsivity score

Table S17.

*Replication Sample: Brown-Forsythe Test Results – AQ and CPRS Subscales by Factor Mixture Modelling Class Assignment*

| Subscale | *F** value | *p* value |
| --- | --- | --- |
| *AQ* |  |  |
| AQ_Soc | *F*(1, 170.005) = 99.782 | < .001** |
| AQ_Att_Sw | *F*(1, 204.681) = 59.563 | < .001** |
| AQ_Com | *F*(1, 172.720) = 162.246 | < .001** |
| AQ_Imag | *F*(1, 185.211) = 66.976 | < .001** |
| *CPRS* |  |  |
| CADHD | *F*(1, 145.727) = 764.719 | < .001** |
| CCGI | *F*(1, 144.326) = 461.527 | < .001** |
| CDSM_Inatt | *F*(1, 151.738) = 602.401 | < .001** |
| CDSM_Hyp | *F*(1, 140.947) = 474.333 | < .001** |

*Note*. ** = Significant after Benjamini-Hochberg correction for false discovery rate of .001; ­AQ_Soc = AQ Social Skill subscale; AQ_Att_Sw = AQ Attention Switching subscale; AQ_Com = AQ Communication subscale; AQ_Imag = AQ Imagination subscale; CADHD = CPRS ADHD Index; CCGI = CPRS Global Index; CDSM_Inatt = CPRS DSM-IV Inattention score; CDSM_Hyp = CPRS DSM-IV Hyperactivity/Impulsivity score

References

ANDRASZEWICZ, S., SCHEIBEHENNE, B., RIESKAMP, J., GRASMAN, R., VERHAGEN, J. & WAGENMAKERS, E.-J. (2015). An Introduction to Bayesian Hypothesis Testing for Management Research. *Journal of Management,* 41**,** 521-543.

FORNELL, C. & LARCKER, D. F. (1981). Evaluating Structural Equation Models with Unobservable Variables and Measurement Error. *Journal of Marketing Research,* 18**,** 39-50.

GREENBAUM, P., BOCA, F., DARKES, J., WANG, C.-P. & GOLDMAN, M. (2005). Variation in the Drinking Trajectories of Freshmen College Students. *Journal of consulting and clinical psychology,* 73**,** 229-238.

HAIR, J. F. (2014). *Multivariate data analysis*: Harlow : Pearson Education Limited.

JEFFREYS, H. (1961). *Theory of Probability,* Oxford: Oxford University Press.

LO, Y., MENDELL, N. R. & RUBIN, D. B. (2001). Testing the number of components in a normal mixture. *Biometrika,* 88**,** 767-778.

MCLACHLAN, G. J. (1987). On Bootstrapping the Likelihood Ratio Test Statistic for the Number of Components in a Normal Mixture. *Journal of the Royal Statistical Society: Series C (Applied Statistics),* 36**,** 318-324.

NYLUND, K. L., ASPAROUHOV, T. & MUTHÉN, B. O. (2007). Deciding on the Number of Classes in Latent Class Analysis and Growth Mixture Modeling: A Monte Carlo Simulation Study. *Structural Equation Modeling: A Multidisciplinary Journal,* 14**,** 535-569.
